# Improved VSV-Ebola-GP booster vaccination approach promotes antibody affinity maturation and durable anti-Ebola immunity in humans

**DOI:** 10.1038/s41590-026-02459-w

**Published:** 2026-03-19

**Authors:** Surender Khurana, Olivia Posadas, Lela Kardava, Elizabeth M. Coyle, Supriya Ravichandran, Gabrielle Grubbs, Rosemary McConnell, Nadine Rouphael, Guillaume Poliquin, Joanne M. Langley, Eric Chu, Dean A. Follmann, Xiaoran Shang, Yuxing Li, Susan Moir, Richard T. Davey

**Affiliations:** 1 Division of Viral Products, Center for Biologics Evaluation and Research (CBER), FDA, Silver Spring, MD USA; 2 Laboratory of Immunoregulation, National Institute of Allergy and Infectious Diseases, National Institutes of Health, Bethesda, MD USA; 3 Critical Care Medicine, Clinical Research Center, National Institutes of Health, Bethesda, MD USA; 4 Hope Clinic of the Emory Vaccine Center, Emory University, Atlanta, GA USA; 5 Public Health Agency of Canada, Children’s Hospital Research Institute, Winnipeg, Manitoba Canada; 6 Canadian Center for Vaccinology, IWK Health Centre and Nova Scotia Health Authority, Dalhousie University, Halifax, Nova Scotia Canada; 7 Biostatistics Research Branch, National Institute of Allergy and Infectious Diseases, National Institutes of Health, Bethesda, MD USA; 8 Department of Microbiology and Immunology, Center for Biomolecular Therapeutics, University of Maryland School of Medicine, Baltimore, MD USA; 9 Institute for Bioscience and Biotechnology Research, Rockville, MD USA

**Keywords:** Live attenuated vaccines, Live attenuated vaccines

## Abstract

Short-term boosting of the currently licensed rVSV∆G-ZEBOV-GP vaccine does not generate lasting antibody respones to Ebola virus (EBOV), prompting interest in strategies that elicit more durable immunity. Here, we elucidated the longitudinal humoral immune repertoire over 3 years following prime and boost rVSV∆G-ZEBOV-GP vaccinations administered 18 months apart in healthy adults compared to participants randomized to no boost. This delayed booster vaccination induced long-lasting EBOV-neutralizing antibodies that persisted up to 36 month at levels similar to peak titers after a single dose. Phage display libraries, expressing linear and conformational epitopes of EBOV glycoprotein (GP), demonstrated a highly diverse and durable antibody epitope repertoire following prime boost vaccination. Delayed booster vaccination recalled memory B cells, promoted anti-GP IgG class switching and induced antibodies specific to GP with Fcγ receptor interaction and functional antibody-dependent cellular cytotoxicity and phagocytosis. The 18-month interval led to 13-fold higher antibody affinity maturation than a single dose, maintained up to 36 months. Overall, delayed rVSV∆G-ZEBOV-GP booster vaccination promoted a diverse, stronger, durable, predominant IgG, highly affinity-matured antibody response to GP.

## Abstract (web summary)

A delayed booster of Ebola vaccine induces a stronger and durable antibody response with higher affinity maturation than a single dose, providing long-lasting immunity and enhancing the ability to prevent and control Ebola disease.

## Main

Ebola virus disease (EBOD) is a severe and often fatal illness caused by the highly pathogenic Ebola virus (EBOV), a member of the Filoviridae family. In the 2013–2016 Western African outbreak, there were more than 28,000 confirmed human cases and over 11,000 deaths (with an overall case-fatality rate of 40%)^1^. Several Ebola vaccines tested in animal models have protected against lethal EBOV challenge and have been evaluated in human clinical trials^2–9^. An open-label, cluster-randomized ring vaccination trial (called Ebola ça Suffit!) was conducted in Guinea during the Western Africa outbreak that randomized clusters to immediate versus delayed immunization and established the clinical efficacy of the rVSV∆G-ZEBOV-GP vaccine in preventing EBOD^7^. In the United States, ERVEBO, a replication-competent, live, attenuated recombinant vesicular stomatitis virus (VSV) vaccine expressing Ebola Zaire (renamed *Orthoebolavirus zairense*) surface glycoprotein (GP) from the Kikwit 1995 strain (rVSV∆G-ZEBOV-GP) was approved by the US Food and Drug Administration (FDA) in 2019 for single-vaccine dose administration for the prevention of EBOD and is now authorized for persons 12 months of age and older. Given its proven rapid efficacy in acute settings, the rVSV∆G-ZEBOV-GP vaccine has been deployed in response to subsequent EBOV outbreaks. However, EBOV breakthrough infections have been observed despite rVSV∆G-ZEBOV-GP vaccination possibly due to several factors, including waning immunity, comorbidities, viral relapse or exposure to a high viral load in subsequent studies^10–13^. Nonetheless, breakthrough infections were associated with reduced disease severity and decreased mortality by 50–60% compared to unvaccinated infections in the Democratic Republic of the Congo between 2018 and 2020 (refs. ^10,14,15^). The long-term persistence of virus in some survivors and increasing zoonotic spillover events into humans have raised fears that future outbreaks could occur, resulting in severe epidemics^16^. Therefore, the development of an effective vaccination strategy that might provide greater long-term protection against EBOD is a high priority^17^.

The vaccination-induced humoral immune response is important in protecting against EBOD, although cellular immunity can also play a role^18–22^. In previous studies, we showed that single-dose rVSV∆G-ZEBOV-GP vaccination induced EBOV-neutralizing antibodies and/or GP-binding antibodies that were not boosted by a second 20 million-plaque-forming units (p.f.u.) rVSV∆G-ZEBOV-GP vaccine dose when given only 28 days after the first vaccination^8,23^. This prime–boost approach with the booster vaccine given at a short time interval predominantly induced an IgM antibody response that was short lived, declined rapidly within a few months and neither improved the quality of antibodies nor induced minimal antibody affinity maturation. The phenomenon of improved immune quality with extended dosing intervals between prime and boost has been well established in other vaccine platforms, such as severe acute respiratory syndrome coronavirus 2 (SARS-CoV-2) mRNA vaccines, where longer intervals between doses led to enhanced humoral immunity and improved T cell response^24^. Likewise, longer prime–boost intervals with H5N1 influenza DNA vaccines have demonstrated superior B cell responses and broader antibody repertoires^25,26^. To build on these findings and other clinical studies, we performed a follow-up clinical study that investigated the impact of delayed booster vaccination given at 18 months after first vaccination in a phase 2 randomized, controlled clinical trial^27^. The 18-month time interval between prime and boost was chosen based on study constraints and indications that an extended interval for immune cells to return to a resting state may be needed for an effective booster response^25–29^.

In this study, we performed comprehensive longitudinal analysis of the humoral immune repertoire to evaluate the durability and quality of the vaccination-induced immune response over 36 months following primary rVSV∆G-ZEBOV-GP vaccination compared to homologous rVSV∆G-ZEBOV-GP vaccine booster given at month 18 in samples collected from healthy adult volunteers from a randomized phase 2 trial^27^. Quantitative and qualitative analyses were performed to elucidate antibody epitope repertoires using EBOV-GP gene fragment phage display libraries (EBOV-GP GFPDLs), EBOV-GP-specific B cell subsets, Fcγ receptor (FcγR) interactions and surface plasmon resonance (SPR) technology to measure real-time antibody binding kinetics, antibody cross-reactivity, B cell responses, immunoglobulin isotyping, antibody affinity maturation and antibody persistence in the participants of this trial.

## Results

### Delayed booster vaccination with rVSV∆G-ZEBOV-GP at 18 months promotes durable EBOV-neutralizing antibodies

**Fig. 1:**
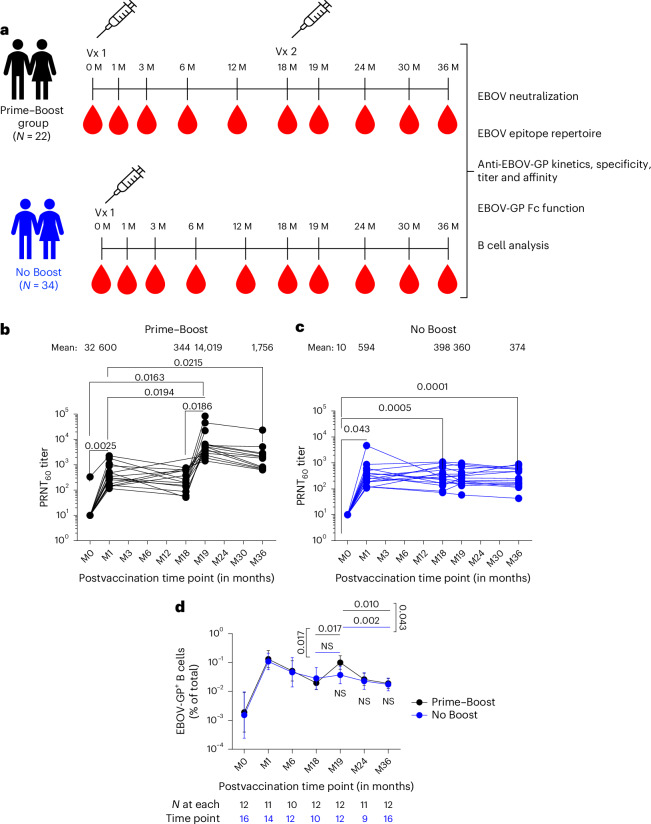
Study design and neutralizing antibodies generated following first and second rVSV∆G-ZEBOV-GP vaccination. **a**, Overview of the cohort with longitudinal serum samples collected from 22 vaccinees in the Prime–Boost group and 34 adults in the prime-only No Boost cohort. Comprehensive antibody profiling of serum samples from both cohorts was performed via assessing EBOV-neutralizing antibodies using PRNT, determining the EBOV-GP antibody epitope repertoire by GFPDLs, characterizing EBOV Kikwit and Makona strain GP binding antibody kinetics using SPR, assessing IgG–FcγR interactions using a Luminex and functional assay, and performing B cell analyses using flow cytometry; M, month. **b**,**c**, Neutralizing antibody titer (PRNT_60_) data for VSV∆G-ZEBOV-GP Kikwit strain are shown for the Prime–Boost group (**b**; *n* = 15) and No Boost group (**c**; *n* = 16). **d**, Frequencies of EBOV-GP^+^ B cells are shown as geometric mean with 95% confidence interval for Prime–Boost and No Boost groups. The number of individuals tested is shown below each time point. Differences between time points within each group or between groups were examined for statistical significance by two-sided paired or unpaired *t*-tests, respectively, and statistically significant values (*P* < 0.05) are shown; NS, not significant. Source data

Longitudinal antibody repertoire profiling was performed on pre- and postvaccination serum samples collected at multiple time points for up to 3 years from healthy adults vaccinated with a 20 million-p.f.u. dose of rVSV∆G-ZEBOV-GP^27^ (Fig. 1a and Extended Data Table 1). Serologic analyses were performed on 22 individuals who received a prime vaccination at month 0, followed by a booster vaccination at 18 months (Prime–Boost group), whereas 34 individuals only received a single vaccine dose at month 0 (No Boost group). Samples were tested for EBOV-neutralizing antibodies by plaque reduction neutralization testing (PRNT), polyclonal antibody epitope repertoire of post-vaccination sera by EBOV-GP GFPDLs, GP-binding antibodies, cross-reactivity to Makona GP, anti-GP antibody affinity maturation and antibody isotypes by SPR, EBOV-GP-specific FcγR antibody interaction by bead assay and functional assay, and EBOV-GP-specific B cell responses by multiparameter spectral flow cytometry.

**Extended Data Fig. 1:**
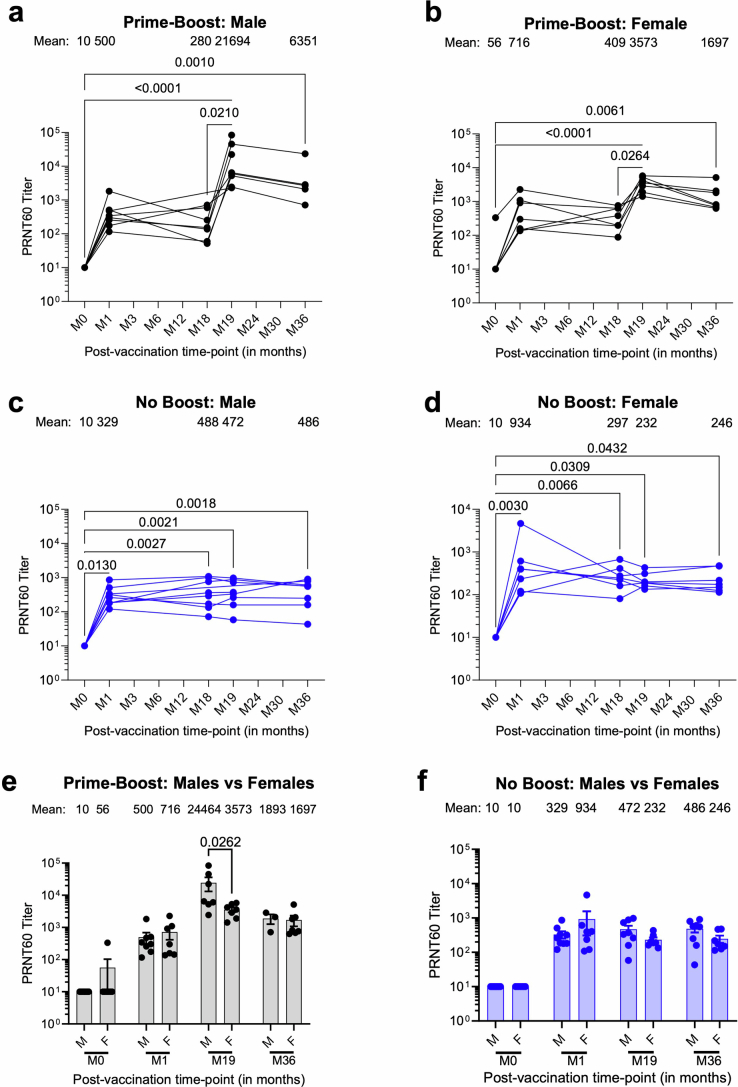
Neutralizing antibodies generated following first and second rVSVΔG-ZEBOV-GP vaccination in males and females. Neutralizing antibody titer (PRNT60; serum titer to reduce 60% plaques) against rVSV∆G-ZEBOV-GP-Kikwit strain are shown for the prime-boost group (**a**-**b**; n = 15) and no-boost group (**c**-**d**; n = 23) by males (**a** & **c**) and females (**b** & **d**). Comparison of PRNT60 titers between males and females at each time-point for prime-boost group (**e**) and no boost group (**f**) are shown. Mean neutralization titers are shown for each time-point above the graph. Data are presented as mean values +/- standard error of the mean. Differences between time-points within each group (**a**-**d**) or between groups (**e**-**f**) were examined for statistical significance by two-sided paired or unpaired t-tests, respectively, and statistically significant values (p values < 0.05) are shown. Source data

The samples were tested for neutralizing antibodies against rVSV∆G-ZEBOV-GP vaccine virus by PRNT to determine the serum titer to reduce 60% of plaques (60% neutralization titer; PRNT_60_)^30^. Very low neutralizing antibodies (PRNT_60_ ≤ 1:20) to rVSV∆G-ZEBOV-GP vaccine virus were observed in prevaccination samples of all but one individual that were near the limit of detection for the PRNT (Fig. 1b,c). Primary rVSV∆G-ZEBOV-GP vaccination induced significantly higher neutralization titers (mean PRNT_60_ of 600) that peaked at 1 month after vaccination than prevaccination titers across both groups. There was no significant difference between the groups. Neutralizing antibodies declined over time to reach mean PRNT_60_ titers of 344–398 at 18 months after primary vaccination across study cohorts. A booster rVSV∆G-ZEBOV-GP vaccination given at 18 months after the primary vaccination generated 23-fold higher neutralizing antibody titers (mean PRNT_60_ titer of 14,019) than those induced at 1 month after primary vaccination (Fig. 1b). Moreover, postbooster neutralizing antibodies were 5.1-fold higher (mean PRNT_60_ of 1,756) at 18 months after second vaccination (36-month time point) than neutralization titers at 18 months after the first vaccination (PRNT_60_ of 344) in the Prime–Boost group. PRNT_60_ titers showed similar kinetics and magnitudes in both men and women after the first and second vaccinations (Extended Data Fig. 1).

**Extended Data Fig. 2:**
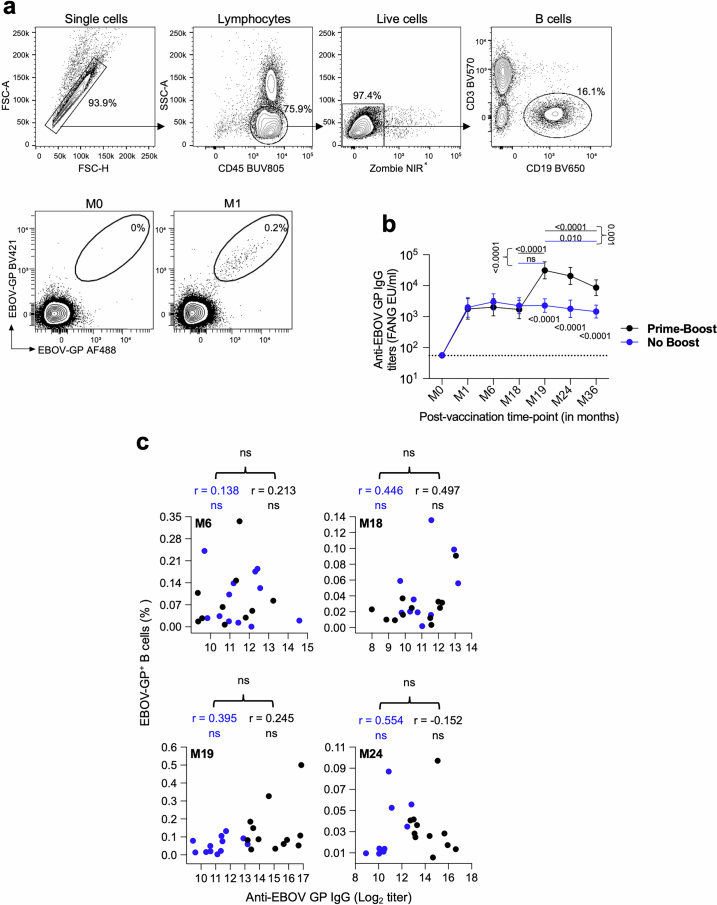
EBOV-GP B cell and antibody analyses. (**a**) Representative binding of EBOV-GP tetramers on two different fluorochromes to B cells is shown with gating strategy (CD45^+^CD3^−^CD19^+^) prior to (M0) and after (M1) primary vaccination with rVSV∆G-ZEBOV-GP. (**b**) Titers of anti-EBOV-GP IgG binding antibodies are shown as geometric mean with 95% CI for prime-boost and no-boost groups. The individuals tested at each time-point are those shown in Fig. 1d. Differences between time-points within each group (increase M18 to M19: p < 0.0001 for prime-boost and not significant (ns) for no-boost group; decrease M24 to M36: p < 0.0001 for prime-boost and p = 0.01 for no-boost group) or between groups (p < 0.0001 at M18, M24 and M36; p < 0.0001 slope increase M18 to M19 and p = 0.001 slope decrease M24 to M36 prime-boost versus no-boost groups) were examined for statistical significance by two-sided Student paired or two-sample t-tests, respectively. (**c**) Correlations between mean anti-EBOV GP IgG antibody titers and frequencies of EBOV-GP^+^ B cells shown for each vaccine group at M6, M18, M19 and M36 time-points were determined using two-sided Pearson’s method. A Fisher’s z-transformation was used to compare the correlation coefficients (r) between the two groups, and numbers or ns shown represent values that were statistically significant (p values < 0.05) or not, respectively. Source data

EBOV-GP-specific B cell responses were evaluated in the limited number of individuals from whom peripheral blood mononuclear cells (PBMCs) were collected, stored and available for analysis (Fig. 1d). The analyses were performed by flow cytometry using tetramers of EBOV-GP conjugated to two different fluorochromes after verification of specificity (Extended Data Fig. 2a). Although frequencies of EBOV-GP^+^ B cells rose from a mean of 0.005% at baseline (month 0) to 0.19% at 1 month after primary vaccination for both groups combined, there was a moderate effect of the booster vaccine between the two groups. This was despite a significant increase in frequency for the boosted individuals between month 18 and month 19 time points (Fig. 1d) and a significant effect of the booster vaccine on anti-EBOV-GP IgG titers, consistent with data reported for the entire cohort^27^ (Extended Data Fig. 2b). The slopes of increase at the month 18-to-month 19 time interval and decrease at the month 19-to-month 36 time interval, respectively, were significant for the Prime–Boost group compared to the No Boost group. Collectively, these data show that although a single dose of the rVSV∆G-ZEBOV-GP vaccine led to equally robust antibody and B cell responses, the booster dose at month 18 induced strong and sustained neutralizing antibodies, yet B cell responses were more modest and short lived in systemic circulation.

### Diverse antibody epitope repertoire following first and second vaccination with rVSV∆G-ZEBOV-GP

**Fig. 2:**
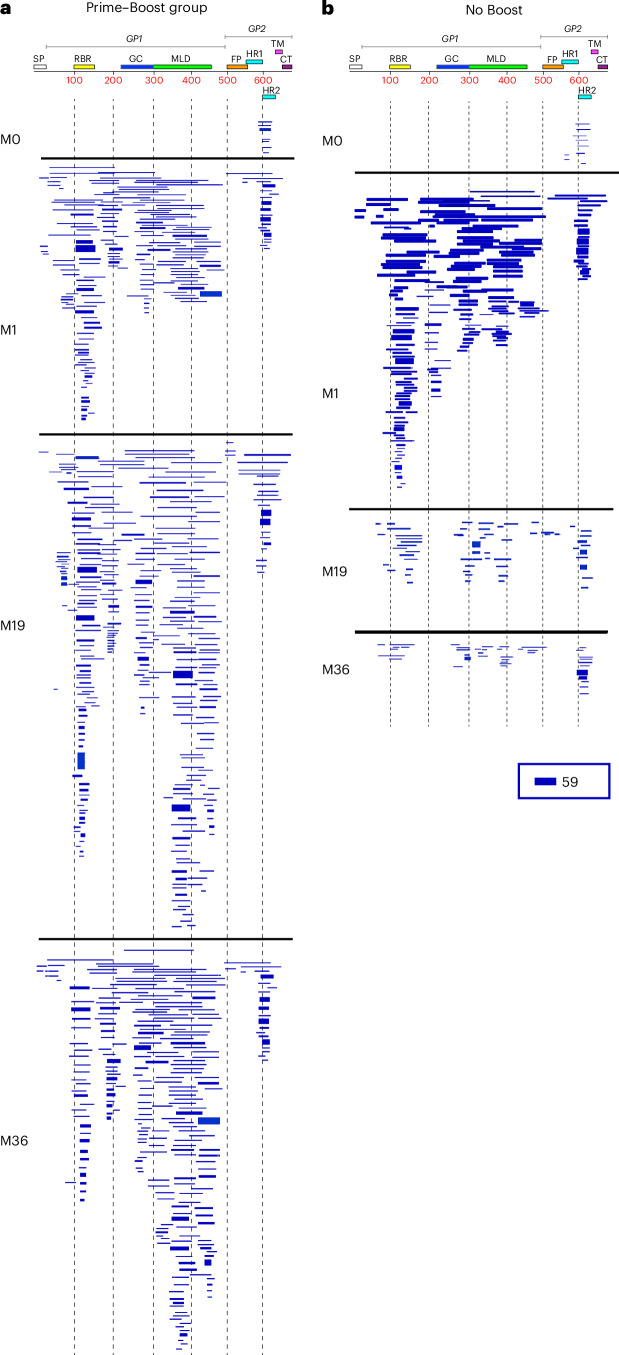
Antibody repertoires elicited following first and second rVSV∆G-ZEBOV-GP vaccination in adults. Schematic alignment of the peptide sequences recognized by sera before (prevaccination (month 0)) or at different time points (1 month, 19 months and 36 months) following rVSV∆G-ZEBOV-GP vaccination in the Prime–Boost group (**a**) or No Boost group (**b**), identified by selection with EBOV-GP GFPDLs. The amino acid designation is based on the complete EBOV Kikwit GP sequence, and GP1 and GP2 subdomains are shown. The GP RBR is depicted as yellow, and the mucin-like domain (MLD) is shown in light green. Other GP domains such as signal peptide (SP), glycan cap (GC), fusion peptide (FP), heptad repeat 1 (HR1), heptad repeat 2 (HR2), transmembrane (TM) and cytoplasmic tail (CT) are depicted. Bars indicate identified inserts in the GP sequence. A graphical distribution of representative clones with a frequency of ≥2, obtained after affinity selection, is shown. The horizontal position and the length of the bars indicate the peptide sequence displayed on the selected phage clone to its homologous sequence in the EBOV-GP sequence on alignment. The thickness of each bar represents the frequency of repetitively isolated phage, with the scale shown below the alignment.

The polyclonal antibody epitope repertoire of pre- and postvaccination serum samples following rVSV∆G-ZEBOV-GP vaccination were elucidated using EBOV-GP GFPDLs containing a random distribution of sequences and fragment sizes ranging from 50 to 1,000 bp in length of the gene encoding GP from EBOV Kikwit strain with >10^7^ unique phage clones, expected to display all possible linear and conformational epitopes, as described before^23,31^. Previous studies for epitope mapping of monoclonal antibodies (mAbs) and adsorption studies using postvaccination polyclonal sera provided support for using the EBOV-GP GFPDLs to define polyclonal antibody repertoires in human sera^23,31^. Serum samples within each group were pooled from each individual that underwent collection before vaccination (month 0), at 1 month after the first vaccination, at 19 months (1 month after the second vaccination in the boost group) and at month 36 (18 months after the second vaccination in the boost group) to determine overall antibody epitope repertoires (Fig. 2).

Prevaccination serum bound very few phages (<100) that mapped to the C-terminal domain of GP2 in both groups (Fig. 2a,b). At 1 month following primary rVSV∆G-ZEBOV-GP vaccination, the number of bound phages was higher in both groups (1.04 × 10^6^ and 1.17 × 10^6^ phages, respectively). Sequencing of GP fragments expressed by phages bound with sera following the first vaccination in both cohorts showed high frequency of bound phages displaying both small and large fragments mapping across the entire GP1 head domain and the C terminus of the EBOV GP2 domain. Within the GP1 domain, post-first vaccination antibodies recognized several small and large immunodominant epitopes in the N-terminal half of EBOV-GP mapping to the receptor binding region (RBR), the region between the RBR and the glycan cap domain, and the glycan cap and mucin domain. In GP2, the selected phage clones mapped primarily to the heptad repeat domain 2.

In the No Boost group, at 19 months after the first vaccination, the bound phage titers declined appreciably such that only 783 phage clones were recognized that mapped to the RBR, glycan cap and mucin domain in GP1 and the C-terminal domain of GP2 (Fig. 2b). By 36 months, only 262 clones were identified that primarily mapped to the C terminus of GP2 and some interspersed sites in GP1.

Importantly, a second rVSV∆G-ZEBOV-GP vaccination given at 18 months after the first vaccination boosted the number of EBOV-GFPDL-bound phages (3.95 × 10^6^ phages) by 3.7-fold relative to the first vaccination (Fig. 2a). The antibody epitope profiles were diverse, with a prominent increase in antibodies specific to the RBR and glycan cap and antibodies recognizing large sequences in the mucin-like domain of GP1. Interestingly, even after 18 months following the second vaccination, the antibody repertoire diversity across GP1 and GP2 persisted, and phage titers (1.98 × 10^6^ phages) only declined twofold by 36 months compared to month 19.

### Antibody binding to EBOV Kikwit and Makona GP

**Fig. 3:**
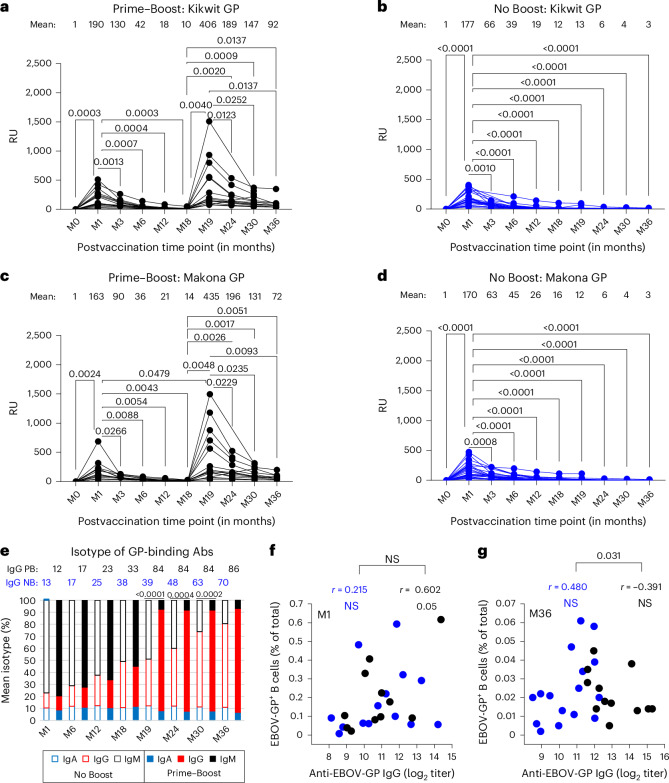
Anti-EBOV-GP and B cells following rVSV∆G-ZEBOV-GP vaccination. **a**–**d**, Serum samples collected at different time points from adults vaccinated before and at different time points after rVSV∆G-ZEBOV-GP vaccination (in months) were analyzed for total antibody binding to purified mature GP from EBOV Kikwit (**a** and **b**) and EBOV-Makona (**c** and **d**) strains for the ‘Prime–Boost’ group (**a** and **c**; *n* = 15) and ‘No Boost’ group (**b** and **d**; *n* = 24) by SPR. Individuals were vaccinated intramuscularly on day 0 in both groups and boosted at 18 months in the Prime–Boost group (**a** and **c**). Total EBOV-GP-binding antibodies are represented in SPR RU for each individual at all time points. Mean RU is shown for each time point above the graph. Differences between time points within each group were examined for statistical significance by two-sided paired *t*-test, and statistically significant values (*P* < 0.05) are shown. **e**, Isotyping of human serum antibodies bound to GP of the EBOV Kikwit strain at different time points following rVSV∆G-ZEBOV-GP vaccination as measured by SPR. The RU for anti-GP-bound IgM/IgG/IgA was divided by the total RU for total bound IgM + IgG + IgA combined for each serum sample and is represented as a percentage. Mean percent IgG is shown at different time points for each cohort: No Boost (NB) and Prime–Boost (PB). **f**,**g**, Correlations between mean anti-EBOV-GP IgG titers and frequencies of EBOV-GP^+^ B cells determined using two-sided Pearson’s method for each vaccine group at month 1 (**f**) and month 36 (**g**) time points. A Fisher’s *z* transformation was used to compare the correlation coefficients (*r*) between the two groups, and numbers shown represent values that were statistically significant (*P* < 0.05). Source data

As the EBOV-GP GFPDL analyses were performed on pooled serum samples from each group, we performed quantitative analyses of sera from each individual at all time points with the recombinant GP derived from the vaccine-homologous Kikwit strain as well as the 2014-Makona strain responsible for the largest EBOV outbreak to date (Fig. 3a–d). The SPR-based assay measures the total EBOV-GP binding antibody titer independent of a secondary antibody and determines the total combined antibody binding of all isotypes (IgM + IgG + IgA) in the polyclonal sample to EBOV-GP captured on a sensor chip as resonance units (RU).

Prevaccination sera (month 0) did not show antibody binding to GP on either the 1995-Kikwit (Fig. 3a,b) or 2014-Makona strain (Fig. 3c,d) in either of the cohorts. At 1 month after first rVSV∆G-ZEBOV-GP vaccination, all samples from both groups reacted with GP from Kikwit and Makona strains, with mean titers ranging between 163 and 190 RU, and no significant difference among either of the two vaccine groups or between the two GPs was observed. Subsequently, the total antibody reactivity after the first vaccination decreased over time, with a steeper decline in the first 6 months, such that most of the vaccinated individuals (>80%) at the 6-month time point showed very low anti-GP binding levels to both Kikwit and Makona GPs.

Surprisingly, the second rVSV∆G-ZEBOV-GP vaccination given at 18 months boosted 2.1-fold higher anti-Kikwit GP and 2.6-fold higher antibody titers to Makona GP than anti-GP binding titers at peak (month 1) after the first vaccination (Fig. 3a,c). This contrasts with the previous study where a second vaccination given only 28 days after first vaccination did not improve the anti-GP antibody response^23^. Over time, anti-GP antibody titers also declined after the second vaccination; however, these antibody titers at 18 months after second vaccination (month 36; mean RU of 72–92) were still at least fivefold higher than those observed at 18 months after first vaccination (month 18; mean RU of 10–14) against the GPs of both Kikwit and Makona strains.

**Extended Data Fig. 3:**
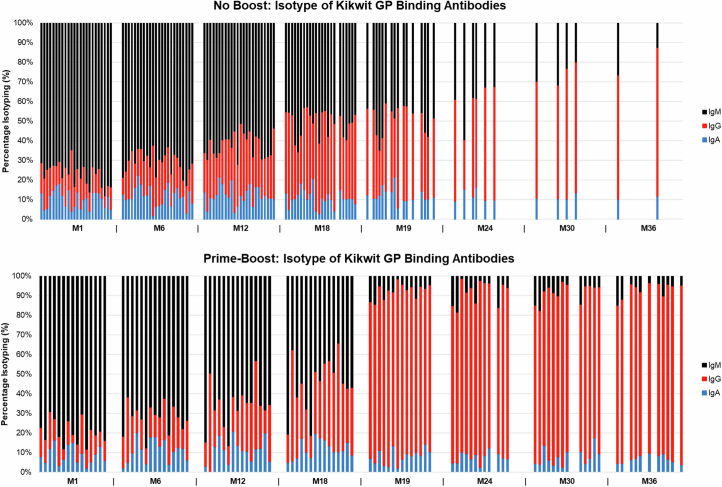
Isotyping of human serum binding to EBOV-GP following rVSV∆G-ZEBOV-GP vaccination. The isotype of serum antibodies bound to GP of EBOV Kikwit strain are shown for serum samples collected at different time points from adults vaccinated with rVSV∆G-ZEBOV-GP vaccine as measured by SPR. The resonance unit for anti-GP bound IgM/IgG/IgA was divided by the total resonance units for total bound IgM+IgG+IgA antibodies combined for each sera and represented as a percentage. Isotyping is only shown for serum samples where total IgM+IgG+IgA antibodies >5 RU in SPR. Several samples in the prime-only ‘no boost’ group at M19 to M36 time-points had titers <5RU. Source data

Isotype analysis of the antibodies binding to GP revealed that most of the GP-binding antibodies at 1 month after primary vaccination were of the IgM isotype (mean of 77–79%), with a smaller representation of IgG (mean of 12–13%) and IgA (mean of 9–10%) in the two cohorts (Extended Data Fig. 3). Over time, these antibodies binding to GP declined such that at 18 months after first vaccination, the anti-GP present consisted of 50–55% IgM, 33–38% IgG and 11–12% IgA in the two groups. At later time points (months 24–36) after the first vaccination, the residual antibodies binding to GP remaining in the sera were IgG, suggesting that most of the antibodies to GP generated early following single rVSV∆G-ZEBOV-GP vaccination were IgM, which are short lived and do not provide long-lasting systemic anti-GP humoral immunity. Notably, a booster vaccination given at 18 months changed the distribution of antibodies binding to GP, with IgG frequency in the serum increasing to 84%, followed by 8% IgA and 7% IgM at 1 month (month 19) after second vaccination (Fig. 3e). This delayed booster-induced anti-GP IgG response was durable up to at least 18 months after second vaccination (month 36), with a mean IgG frequency of 86%.

The analysis revealed that most of the anti-GP antibody response generated early following first vaccination was of the IgM isotype and were short lived, whereas delayed booster vaccination with replicating rVSV∆G-ZEBOV-GP given at 18 months generated a strong long-lasting systemic cross-reactive anti-GP IgG response to different Ebola Zaire (renamed *O. zairense*) strains.

### B cell responses following rVSV∆G-ZEBOV-GP vaccination

**Fig. 4:**
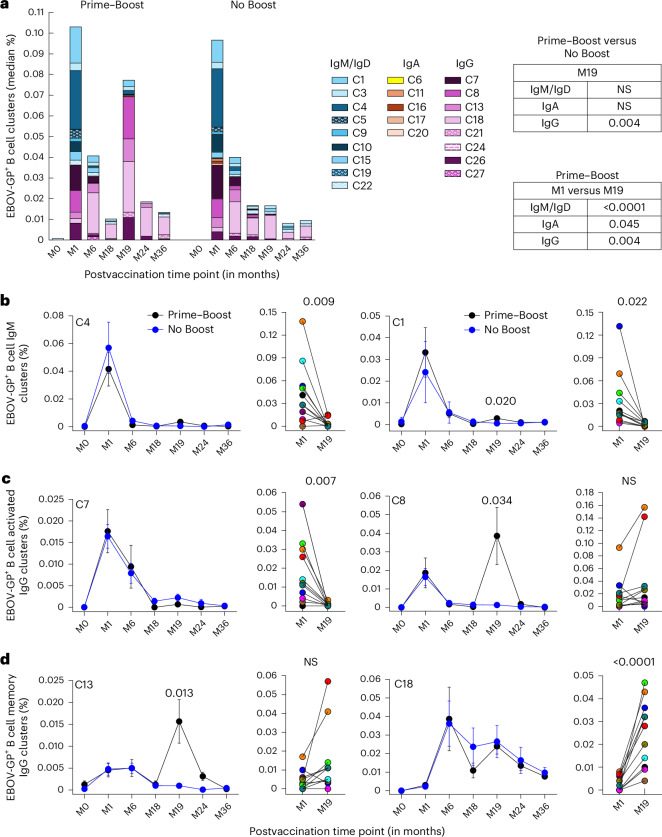
EBOV-GP responses among B cell clusters. **a**, Medians of EBOV-GP^+^ counts within each cluster calculated as a percentage of total CD19^+^ B cells and color coded by immunoglobulin are shown by vaccination group and time point. The color tones are darkest for activated and lightest for quiescent clusters. **b**–**d**, Mean ± s.e.m. and individual EBOV-GP^+^ frequencies for IgM (**b**), IgG activated (**c**) and resting memory (**d**) clusters are shown at the indicated time points by vaccine group and within the Prime–Boost group (*n* = 11), respectively. The number of individuals tested at each time point (**b**–**d**) is shown in Fig. 1d. Differences at specified or all time points (multiple time points in **b**–**d**) between the two groups or within the Prime–Boost group were examined for statistical significance by two-sided Welch’s or paired two-sample *t*-test, respectively. Numbers or NS shown represent values that were statistically significant (*P* < 0.05) or not, respectively, whereas only significant values are shown in graphs with multiple time points. Source data

**Extended Data Fig. 4:**
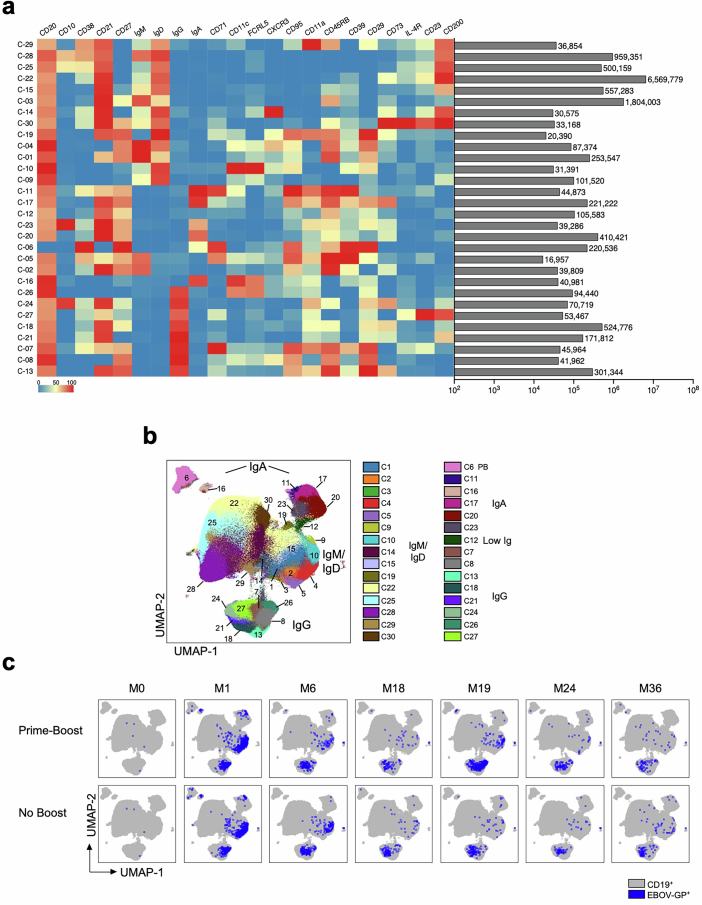
EBOV-GP response among B-cell clusters. (**a**-**b**) Heatmap (**a**) and UMAP (**b**) of unsupervised clustering performed on all B cells with 22 B-cell markers is shown, along with the number of cells in each cluster. (**c**) UMAP in (**b**) overlaid with EBOV-GP^+^ cells by vaccination group and time-point. Source data

Titers of anti-EBOV-GP IgG and frequencies of EBOV-GP^+^ B cells were directly correlated for both groups at month 1 yet were significantly divergent by month 36, whereby the direction continued to be positive for the No Boost group while turning negative after month 19 for the Prime–Boost group (Fig. 3f,g and Extended Data Fig. 2c). High-dimensional analyses of the flow cytometric data were performed to gain further insight into changes in EBOV-GP-specific B cell responses following vaccination. Unsupervised clustering and dimensionality reduction of total B cells from all time points and individuals combined generated 30 distinct clusters and areas on uniform manifold and approximation projection (UMAP) that were defined by immunoglobulin isotype distribution and dominated by naive B cells (Extended Data Fig. 4a,b and Extended Data Table 2). By contrast, the EBOV-GP-specific response was dominated by activated and memory populations that transitioned from predominantly IgM to IgG clusters over time (Extended Data Fig. 4c). This transition was highly significant and accelerated by the booster dose, as reflected by comparing isotypes after primary (month 1) and secondary (month 19) doses within the Prime–Boost group and at month 19 between the two groups (Fig. 4a).

**Extended Data Fig. 5:**
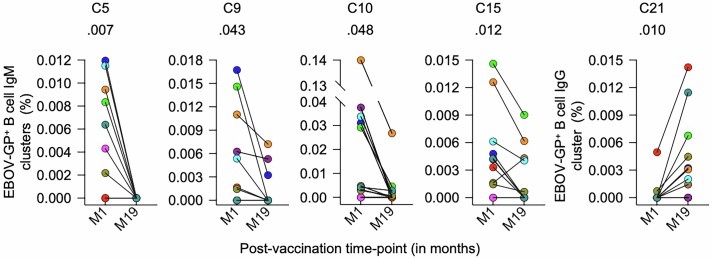
EBOV-GP^+^ clusters compared by time-point in the prime-boost group. Individual EBOV-GP^+^ frequencies for additional IgM and IgG clusters, comparing 1-month responses between primary (M1) and booster (M19) vaccinations. Differences between M1 and M19 within the prime-boost group were examined for statistical significance by a two-sided paired two-sample t-test. Numbers or ns shown represent values that were statistically significant (p values < 0.05) or not, respectively. Source data

Within IgM clusters, activated cluster 4 (C4) and resting memory C1 cells drove the primary EBOV-GP-specific response (Fig. 4a). The contribution of these two clusters, along with other minor IgM clusters, to the total EBOV-GP-specific response was significantly higher after the priming vaccine (month 1) than after the booster vaccine (month 19), although C1 also contributed to the booster response (Fig. 4b and Extended Data Fig. 5). Within IgG clusters, activated C8 (CD21^lo^CXCR3^+^CD95^+^) and the more quiescent relative cells in C13 contributed to the EBOV-GP-specific response after both vaccine doses, whereas activated C7 (CD38^+^CD71^+^CD95^+^) cells only contributed to the primary vaccine dose (Fig. 4c,d). Finally, the main IgG resting memory cluster, C18, and its CD27^lo^ counterpart C21 (Extended Data Fig. 4a) were more strongly induced by the booster dose (month 19) than the primary dose (month 1); however, differences between the two vaccine groups did not reach significance at any of the postbooster vaccine time points (Fig. 4d and Extended Data Fig. 5). Collectively, although these observations are consistent with the antibody data showing a transition from an IgM to an IgG response, the delayed booster vaccine did not induce a strong durable IgG B cell response that is typically driven by activated CD71^+^ phenotypes^32,33^.

### Anti-GP IgG–Fc receptor interactions following rVSV∆G-ZEBOV-GP vaccination

Neutralization by antibodies is often the main mechanism to prevent or reduce viral infection. However, other antibody effector functions mediated by Fc and Fc-domain sensors on adaptive and innate immune cells can act either independently or in combination with neutralization to reduce viral disease or infection. Antibody-dependent cellular phagocytosis (ADCP), antibody-dependent cellular cytotoxicity (ADCC) and the complement system contribute to clearance of viral particles or virus-infected cells through interaction with FcγRI/FcγRIIA, FcγRIIIA and C1q, respectively. Because each FcγR is associated with a specific effector function, determining the FcγR interaction with GP-specific antibody binding induced by rVSV∆G-ZEBOV-GP vaccination can be a good surrogate marker for a potential role of antibody Fc-mediated protection against EBOV.

**Fig. 5:**
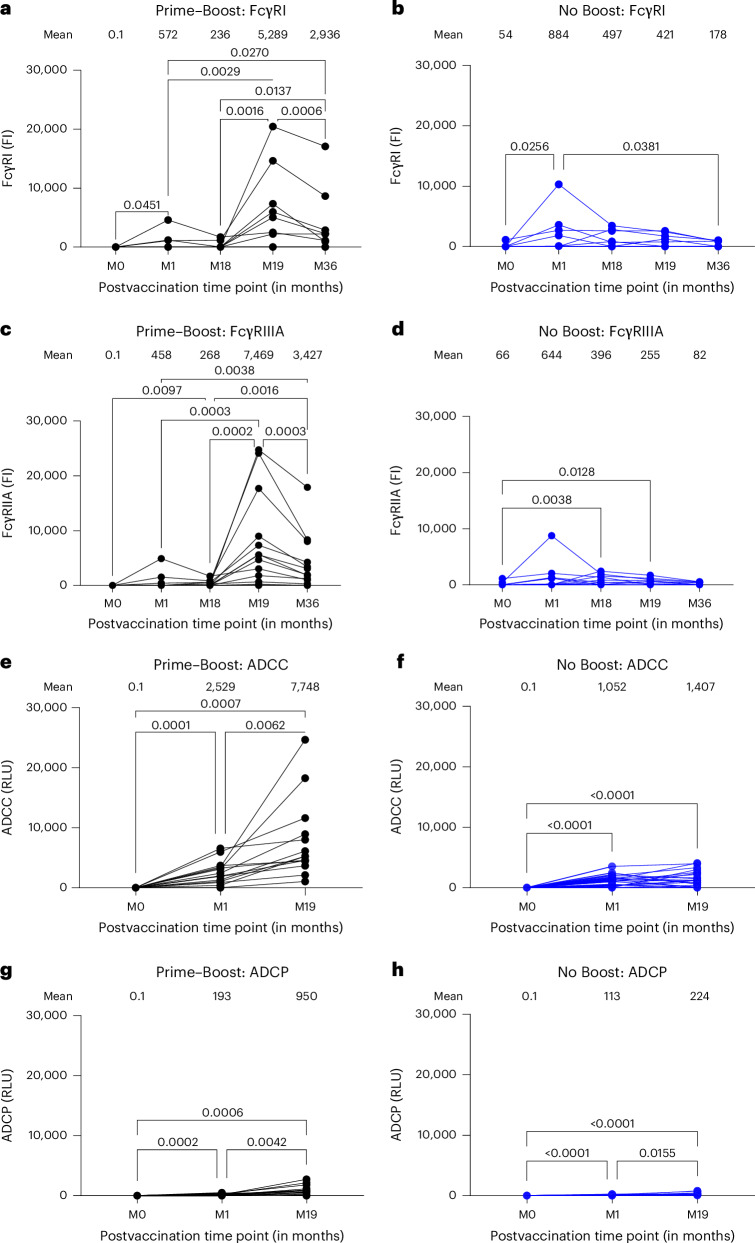
FcγR interaction of rVSV∆G-ZEBOV-GP vaccination-induced serum antibodies. **a**–**d**, Quantification of FcγRI (**a** and **b**) and FcγRIIIA (**c** and **d**) interaction with serum antibodies bound to EBOV Kikwit GP-coupled beads either before (month 0) or at different time points after vaccination as assessed by bead-based assay. Each sample from the ‘Prime–Boost’ group (**a** and **c**; *n* = 15) and ‘No Boost’ group (**b** and **d**; *n* = 23) was run in duplicate, and each symbol represents average fluorescence intensity (FI) of duplicate values. The variation for each sample in duplicate runs was <6%. Mean fluorescence intensity is shown for each time point above the graph. **e**–**h**, Comparison of ADCC and ADCP responses between Prime–Boost and No Boost groups. Panels show mean relative light unit (RLU) values at baseline (month 0), 1 month (month 1) and 19 months (month 19) postvaccination time points above the graph. Differences between time points within each group were examined for statistical significance by two-sided paired Student’s *t*-test, and statistically significant values (*P* < 0.05) are shown. Source data

**Extended Data Fig. 6:**
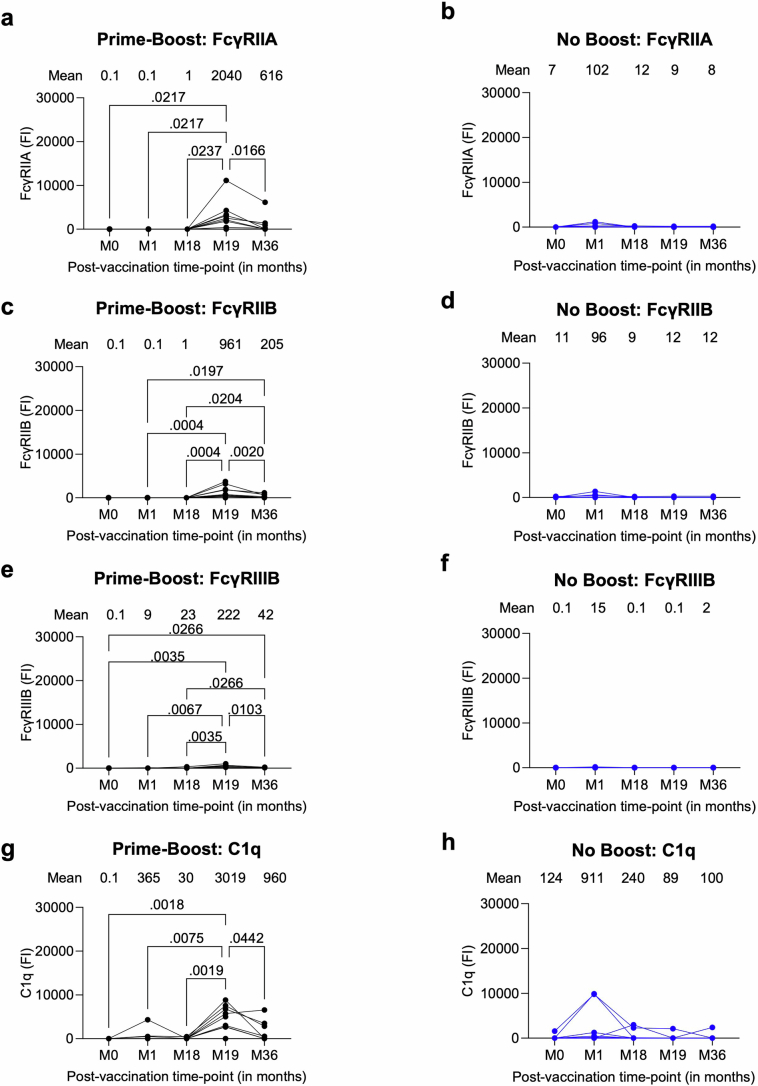
Anti-GP antibody interaction of Fcγ-receptor and C1q following rVSV∆G-ZEBOV-GP vaccination. Binding of pre- and post-vaccination serum antibodies bound to EBOV-Kikwit GP coupled beads with FcγRIIA (**a**-**b**), FcγRIIB (**c**-**d**), FcγRIIIB (**e**-**f**) and C1q (**g**-**h**) proteins either before (M0) or at different time-points post-vaccination (M1: 1-month, M18: 18-months, M19: 19-months and M36: 36-months). Each sample from the ‘prime-boost’ group (**a**, **c**, **e** & **g**; n = 15) and ‘no-boost’ group (**b**, **d**, **f** & **h**; n = 30) was run in duplicate, and each symbol represents average of duplicate values. The level of binding is shown as fluorescent intensity. The variation for each sample in duplicate runs was <6%. Mean fluorescent intensity is shown for each time-point above the graph. Differences between time-points were examined for statistical significance by ANOVA using two-sided Kruskal-Wallis test for multiple comparisons calculated in GraphPad Prism and statistically significant values (p values < 0.05) are shown. Source data

To determine if anti-GP serum IgG generated following rVSV∆G-ZEBOV-GP vaccination has the capacity to mediate various Fc effector functions through engagement with FcRs, polyclonal IgG bound to vaccine-homologous Kikwit GP was evaluated for binding to various FcγRs and C1q by bead-based Luminex assay following vaccination (Fig. 5a–d). At 1 month after first vaccination, anti-GP IgG demonstrated low binding to FcγRI and FcγRIIIA that was boosted 8- to 15-fold by the second vaccination at 1 month (month 19), respectively (Fig. 5a,c). Post-second vaccination-induced FcγRI and FcγRIIIA reactivity remained elevated by five- to sevenfold higher at 18 months (month 36) after second vaccination than peak response (month 1) after first vaccination. Minimal or no binding was observed for FcγRIIA, FcγRIIB, FcγRIIIB and C1q to post-first vaccination anti-GP IgG in either study group (Extended Data Fig. 6). However, second vaccination induced moderate but significantly higher reactivity with FcγRIIA, FcγRIIB and C1q and low reactivity with FcγRIIIB.

**Extended Data Fig. 7:**
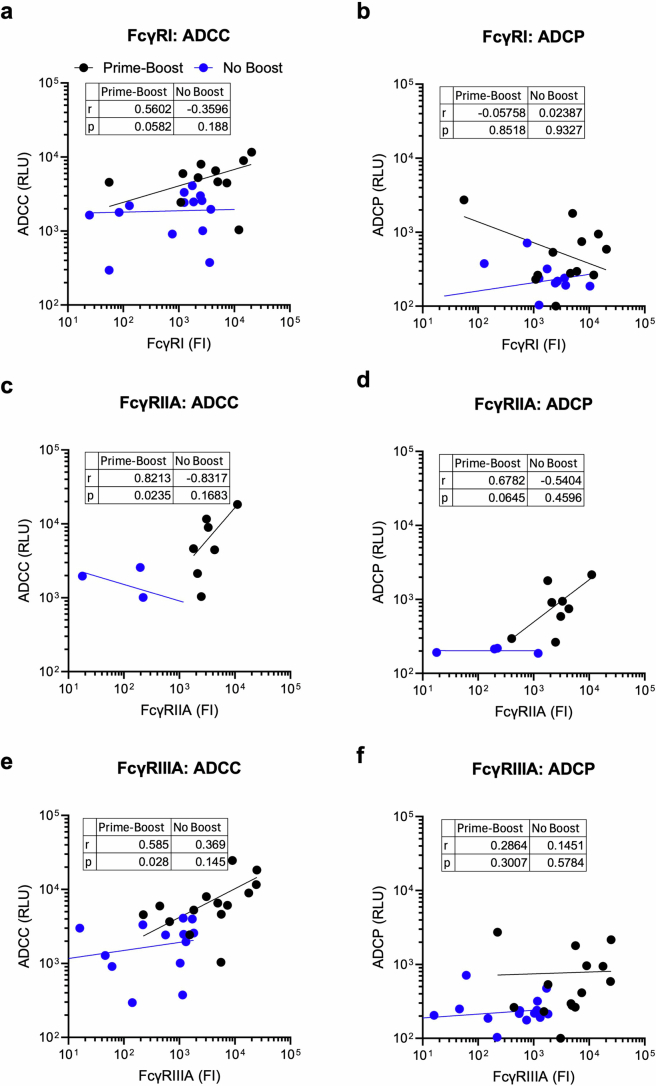
Correlation analysis between Fc gamma receptor binding and functional antibody responses. Scatter plots show the relationship between FcγRI (panels **a**-**b**), FcγRIIA (panels **c**-**d**), and FcγRIIIA (panels **e**-**f**) GP-binding antibodies (x-axis, fluorescence intensity) and ADCC (**a**, **c**, **e**) or ADCP (**b**, **d**, **f**) activity of anti-GP antibodies (y-axis, relative light units) for prime-boost (black) and no-boost (blue) vaccination groups. Data is depicted for 1 month and 19 months post-vaccination serum antibodies. Each data point represents an individual subject’s paired Fc-receptor binding and functional activity measurements. Data is shown only for samples with FcγR binding or functional (ADCC/ADCP) activity above the background signal. Pearson correlation coefficients (r) and p-values (p) are displayed for each group within each panel. Both x and y axes are represented in a log10 scale. Nonlinear regression demonstrated by trendlines was performed in GraphPad Prism. Source data

Functional analysis revealed that in the Prime–Boost group, ADCC activity increased significantly from a baseline (month 0) mean of 0.1 to 2,529 at month 1 and from month 1 to a peak mean of 7,748 at month 19 (Fig. 5e,f). By contrast, there was no increase in the No Boost group between month 1 and month 19. A similar pattern was observed for ADCP, albeit with different kinetics and magnitude. In the Prime–Boost group, ADCP activity increased significantly from a baseline (month 0) mean of 0.1 to 193 at month 1 and further to 950 at month 19 (Fig. 5g,h). The No Boost group showed ADCP activity increasing significantly from a baseline (month 0) mean of 0.1 to 113 at month 1 and 224 at month 19. Although both groups achieved statistical significance from baseline, the Prime–Boost group had 5.5-fold higher peak ADCC and 4-fold higher peak ADCP activities than the No Boost group. This suggests that a Prime–Boost regimen induces a superior antibody effector function, which may contribute to vaccine efficacy. Correlations between FcγR binding and functional responses were performed and revealed distinct patterns relative to ADCC and ADCP activities (Extended Data Fig. 7). For the Prime–Boost group, ADCC activity showed moderate significant correlation with FcγRIIA and FcγRIIIA binding. For ADCP activity, moderate correlation with FcγRIIA binding was observed and minimal with FcγRIA and FcγRIIIA binding, suggesting minimal association between FcγRIA and FcγRIIIA binding and phagocytic activity.

### Delayed booster rVSV∆G-ZEBOV-GP vaccination promotes antibody affinity maturation

**Fig. 6:**
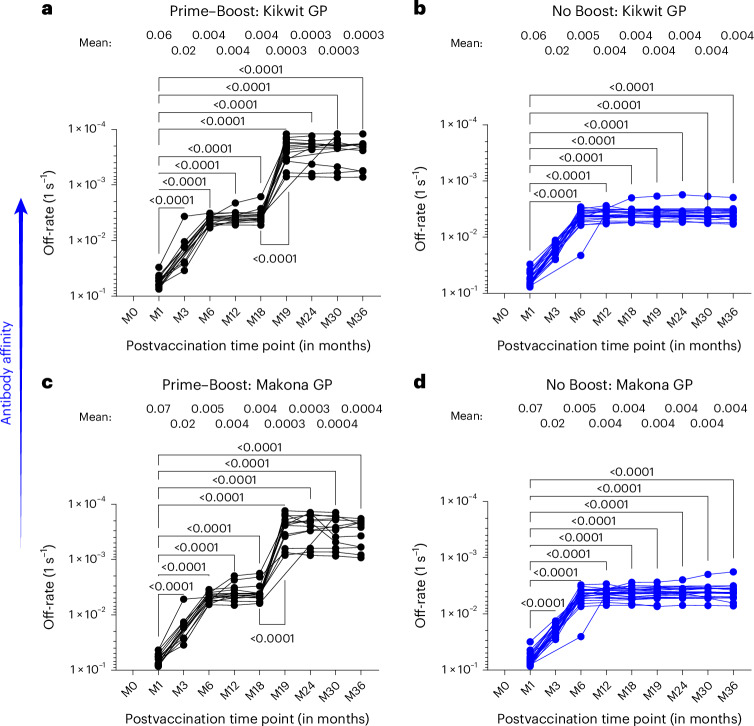
Polyclonal antibody affinity maturation to EBOV-GP following rVSV∆G-ZEBOV-GP vaccination. Antibody affinity of postvaccination serum samples was determined by measuring dissociation kinetics (off-rates) of polyclonal serum antibodies from all individuals against vaccine-homologous EBOV Kikwit GP (**a** and **b**) and Makona GP (**c** and **d**) using SPR. Antibody off-rate constants that describe the fraction of antibody–antigen complexes decaying per second were determined directly for each serum sample from the ‘Prime–Boost’ group (**a** and **c**; *n* = 15) and ‘No Boost’ group (**b** and **d**; *n* = 24) using SPR. Each symbol represents the average of duplicate values. The variation for each sample in duplicate runs was <6%. The mean off-rate for each time point postvaccination is shown above the graph. Differences between time points were examined for statistical significance by two-sided paired Student’s *t*-test, and statistically significant values (*P* < 0.05) are shown. Source data

As noted above, a previous study showed that prime–boost rVSV∆G-ZEBOV-GP vaccination given at 28 days apart did not improve antibody affinity to EBOV-GP^23^. To further investigate the impact of a delayed prime–boost rVSV∆G-ZEBOV-GP vaccination given at 18 months on anti-GP affinity maturation, we determined the dissociation rates (off-rate constants) of postvaccination serum antibody–antigen complexes using SPR, which is independent of antibody concentration and provides a measure of overall avidity of polyclonal antibody binding as previously described^34^. One month following primary vaccination, the off-rates for polyclonal serum antibodies bound to GP were fast (6 × 10^−2^ per s) against GP of both vaccine-homologous Kikwit and Makona strains, indicating weak antibody affinity (Fig. 6). These anti-GP affinities matured over time to peak at ~4 × 10^−3^ per s at 6 months after first vaccination and then plateaued thereafter.

Surprisingly, the delayed second vaccination promoted a faster and significantly stronger antibody affinity maturation with an approximately tenfold higher GP-specific antibody affinity (mean off-rate of 3 × 10^−4^ per s) than those induced after first vaccination that peaked within 1 month following booster vaccination to GP of both strains (Fig. 6a,c). Importantly, these high-affinity antibodies to GP were long lasting and were maintained up to at least 18 months after second vaccination in these prime-boosted vaccinated individuals.

Together, our study demonstrates that delayed rVSV∆G-ZEBOV-GP vaccination promotes a highly diverse, strong, durable, predominantly IgG, highly affinity-matured antibody response to GP.

## Discussion

The US FDA-licensed rVSV∆G-ZEBOV-GP (ERVEBO) vaccine has been routinely used as a single-dose preventative vaccine during EBOV outbreaks. However, both EBOV breakthrough infections and morbidity and mortality due to EBOV infection have been observed despite rVSV∆G-ZEBOV-GP vaccination. Several possible factors may be responsible for this, including inadequate time to mount an immune response between vaccination and exposure, underlying host immunodeficiency, viral relapse and waning immunity following vaccination^10,14,15^.

To improve the durability and quality of the immune response induced by a single-dose vaccine, the role of a second dose of rVSV∆G-ZEBOV-GP vaccine given at a standard 1-month interval after first vaccination was first explored^23^. However, prime–boost vaccination with a 28-day time interval boosted neither binding antibodies to GP nor neutralizing antibodies to EBOV, both of which declined sharply by 3 months after boost vaccination. Based on these observations and findings from other vaccine trials^25,26^, we investigated whether a delayed prime–boost rVSV∆G-ZEBOV-GP Ebola vaccination approach could improve on the immunogenicity induced by the single-dose regimen^27^. Compared to the 28-day prime–boost interval, a delayed 18-month interval was found to induce substantially higher, durable and qualitatively different anti-EBOV antibody titers. Here, we demonstrate that even after 36 months (18 months after second vaccination), the neutralizing antibody response remained significantly higher than at peak (1 month) after first vaccination. Most importantly, a delayed booster vaccine dose promoted a qualitatively superior, IgG isotype-switched, diverse, highly affinity-matured anti-GP antibody response that was sustained up to 36 months after vaccination.

The IgG epitope repertoire defined by EBOV-GP GFPDL analysis demonstrated that booster vaccination given at 18 months generated a diverse epitope profile across the EBOV-GP. This boosted diverse epitope profile persisted up to 36 months after vaccination and spanned large epitopes in the receptor binding domain (RBD), glycan cap and mucin domain that have been shown to contain neutralizing epitopes recognized by clinically relevant neutralizing mAbs like mAb114 or mAb cocktails ZMab, ZMapp and MB-003 (refs. ^23,35–38^). This observation of continued diversity of the epitope profile following booster vaccination may explain the higher neutralization titers observed at 36 months after vaccination in the Prime–Boost cohort. One possible limitation of GFPDL-based assessments is that they are unlikely to detect paratopic interactions that require post-translational modification or rare quaternary epitopes that cross GP protomers. However, binding to properly folded glycosylated EBOV-GP in SPR experiments can overcome these limitations and provide additional insight into the polyclonal anti-GP antibody response.

Remarkably, real-time antibody kinetics of individual postvaccination sera by SPR revealed several unexpected findings associated with the delay in booster vaccination, including a significant increase in cross-reactive anti-GP antibody response, antibody class switching and high antibody affinity maturation. This is in contrast to the findings observed when the prime–boost vaccination was given with just a 28-day interval; in that case, there was limited impact of the second vaccination on any of those parameters^23^. Our findings are consistent with recent studies showing benefits of an increased time interval between doses of SARS-CoV-2 vaccines and observations from earlier clinical trials on the H5N1 influenza vaccine, as well as H7 influenza vaccine studies, all of which demonstrated that increasing the time interval between primary and secondary vaccine doses induced a superior immune response^24–26,28,33,39–41^. In these latter studies, a minimum of 3 months between first and second vaccine doses was found to be optimal for the neutralizing antibody response and antibody affinity maturation. A delayed interval between vaccine doses provides time for cells responding to the primary immune response to return to a resting state, possibly allowing for a more robust recall response to the secondary dose than shorter prime–boost time intervals. This has been suggested from studying SARS-CoV-2, where residual B cell activation from recent breakthrough infection muted the response to the first booster vaccine^29^. In the current study, most of the EBOV-GP-specific responses at month 1, representing the time interval between doses in prior studies, involved B cell clusters with activated phenotypes, whereas at month 18, just before the booster dose here, EBOV-GP^+^ clusters had more resting phenotypes. However, although the antibody response to the delayed rVSV∆G-ZEBOV-GP booster dose was strong and durable, the corresponding B cell response was modest and short lived, failing to reach statistical significance between the groups at any of the post-boost time points. Furthermore, correlations between antibody and B cell responses at month 36 significantly diverged between the two groups, continuing to be positive for the No Boost group while turning negative for the Prime–Boost group. This divergence may suggest that the booster dose preferentially induced plasma cells, whose frequencies have been strongly correlated with antibody titers, over memory B cells. If so, this is different from other vaccines such as influenza and SARS-CoV-2, where recall B cell and antibody responses are both robust^42^. The recall response following the rVSV∆G-ZEBOV-GP booster dose also failed to engage CD71^+^ activated B cells (C7), which have been associated with long-term B cell memory^32^. By contrast, a moderate response to the booster dose was induced among CD21^lo^ activated B cells (C8), which are primed to differentiate into plasma cells^43^. Whether these features of the secondary response and the strong induction of IgM during the primary response explain why the rVSV∆G-ZEBOV-GP vaccine generates strong neutralizing antibodies, yet a weak memory B cell response, remains to be investigated. Sampling at earlier time points after vaccination will be needed to further delineate the role of activated B cells in primary and secondary immune responses to rVSV∆G-ZEBOV-GP vaccine.

In addition to virus-neutralizing antibodies, IgG–FcR interactions on various cell types can provide protection from EBOD following virus infection, as was shown for both neutralizing and non-neutralizing mAbs^44,45^. The bead-based assay for IgG Fc binding to FcγR strongly correlates with Fc-mediated functions like ADCC and ADCP, as demonstrated in previous studies^46,47^. Single rVSV∆G-ZEBOV-GP vaccination induced antibodies to GP that showed minimal FcγR or C1q binding. Importantly, the delayed prime–boost rVSV∆G-ZEBOV-GP vaccination induced antibodies specific to GP that showed strong FcγRI and FcγRIIIA reactivity, suggesting potential for these anti-GP IgG to mediate ADCP and ADCC activities, respectively. This was confirmed in functional activity assays where the prime–boost vaccination strategy was found to be superior in magnitude and durability to no boost in eliciting both ADCC and ADCP responses. These data suggest that the secondary stimulation from the booster dose amplifies the functionality of antibody responses beyond what can be achieved with primary vaccination alone. Correlations were particularly strong and significant between ADCC activity and FcγRIIA binding. This finding is biologically relevant given that FcγRIIA is a key receptor involved in ADCC mediated by natural killer cells and other effector cells^47^. The weaker correlations observed for ADCP across all receptor types suggest that phagocytic activity may be influenced by additional factors beyond simple receptor binding, such as immune complex formation, or other immunological parameters not measured here. Nonetheless, the consistently stronger correlations in the Prime–Boost group than in the No Boost group indicate that the booster vaccination not only enhances the magnitude of responses but also tightens the relationship between receptor binding and effector function. Durability of anti-GP IgG with strong FcγR binding activity following delayed prime–boost vaccination suggests that it should be feasible to generate long-lasting anti-GP IgG that might potentially ameliorate EBOD using an appropriate time-interval-based prime–boost vaccination approach with current EBOV vaccines.

One of the most remarkable findings of our study is the observation of strong antibody affinity maturation against EBOV-GP following a 1.5-year interval for the rVSV∆G-ZEBOV-GP prime–boost vaccination. The 13-fold higher antibody affinity following delayed booster vaccination than prime-only antibody affinity was sustained at least up to 36 months in the Prime–Boost group and is at the high end of polyclonal antibody affinity (off-rate of 10^−4^ per s) in polyclonal samples observed following vaccination with several viral vaccines^34,48,49^. This differs from the previous study where the prime–boost vaccination administered at 28 days showed minimal anti-GP antibody affinity maturation. Antibody affinity maturation requires germinal center interaction between antigen-specific follicular helper T cells and B cells in lymph nodes^50,51^. Pre-existing anti-GP antibody or anti-VSV vector responses at the time of booster vaccination can attenuate replication of live, replication-competent rVSV∆G-ZEBOV-GP vaccine and limit GP antigen expression. This may interfere with effective germinal center formation and B cell selection, thereby dampening the quality of the immune response (including antibody class switching, affinity maturation and development of durable humoral immunity), as observed in prior studies^23,25,26^. By allowing sufficient time for vector immunity to wane and immune effectors to return to baseline, the extended 18-month interval before boosting likely enabled more efficient vector replication and antigen presentation. This, in turn, resulted in a more robust recall response characterized by significantly enhanced antibody affinity maturation and prolonged antibody durability^25,26,39,40^. Therefore, our findings suggest that it may be important to use unbiased technologies and develop appropriate assays to advance the understanding of postvaccination antibody responses and help guide evaluation and development of effective vaccination strategies against EBOV and other human pathogens. In this regard, one of the most important aspects of an effective vaccine is to induce antibody affinity maturation using real-time assays like SPR that do not rely on antibody isotype. Real-time kinetic measurements by SPR can quantify all antigen-binding antibodies, including IgM, IgA and IgG, from polyclonal sources and do not rely on the use of chaotropic agents (for example, urea or thiocyanate in an enzyme-linked immunosorbent assay (ELISA)-based avidity assay) that can denature/reduce EBOV-GP or other antigens^34,52,53^. Moreover, SPR has the additional advantage of directly detecting and measuring serum antibodies in minutes, avoiding the long incubation/washing/detection steps of ELISAs, reducing complexity and variability and thereby providing a more accurate characterization of polyclonal antibody avidity^54,55^.

Study limitations include the inability to obtain matching PBMC and serum samples from all individuals due to logistical differences at study sites because of deployments and other restrictions during the COVID-19 pandemic. Nonetheless the findings in the few individuals having matching samples were internally consistent and reached similar overall conclusions from those derived from the complete dataset. Also, these results were generated in healthy individuals residing in high-income countries who were at potential occupational risk of EBOV exposure. Ideally, these studies should be expanded to a larger cohort that includes individuals residing in EBOV-endemic African regions. Moreover, B cell receptor sequencing of EBOV-GP^+^ B cells after a single- versus two-dose immunization would be helpful to confirm the antibody affinity maturation observed in serum samples. Additionally, the study did not evaluate antigen-specific T cell responses, which may complement humoral immunity and contribute to protection against EBOV. However, cellular immunity against both EBOV-GP and VSV vector was reported following both primary and booster vaccination with rVSV∆G-ZEBOV-GP in an earlier study^56^. Finally, future studies should also consider whether an interval shorter or longer than 18 months between vaccinations may further enhance and sustain humoral and cellular immunity against EBOV.

In summary, the main findings of our study are (1) booster rVSV∆G-ZEBOV-GP vaccination at an 18-month interval induced long-lasting EBOV-neutralizing antibodies that persisted up to 36 months (18 months after booster) at levels similar to peak neutralizing antibodies after single vaccination; (2) a highly diverse and durable antibody epitope repertoire was generated following prime–boost vaccination; (3) delayed booster vaccination recalled a weak yet distinct B cell response that promoted anti-EBOV-GP IgG class switching and plasma cell formation; (4) booster vaccination, but not primary vaccination, induced antibodies specific to GP that demonstrated durable FcγR interactions; and, importantly, (5) booster vaccination administered at an 18-month interval promoted 13-fold higher antibody affinity maturation than single vaccination and was sustained up to 36 months in the study participants. These findings highlight the potential of optimized prime–boost strategies with live vectored vaccines to elicit long-lived, high-quality humoral immunity in humans. This could have significant implications for further predeployment immunization strategies and the evaluation of EBOV vaccines. Such knowledge can be used to inform preventative vaccination strategies in high-risk groups, such as health care workers and frontline workers, and in people living in endemic areas, with the ultimate goal of reducing cases, hospitalizations and deaths and thereby enhancing our ability to prevent and better control EBOD outbreaks.

## Methods

### Study samples

This randomized, placebo controlled, prime–boost, phase 2 clinical trial was performed in healthy adults >18 years old at risk for potential occupational exposure to EBOV, as described before^27^ (ClinicalTrials.gov ID: NCT02788227). The rVSV∆G-ZEBOV-GP vaccine consisting of the rVSV Indiana strain, with the GP of the ZEBOV Kikwit 1995 strain replacing the gene encoding the VSV envelope GP, was administered at 20 million p.f.u. in the form of a 1-ml injection at study start (month 0), followed by 1:1 randomization at month 18 to receive either a homologous booster dose (Prime–Boost group) or no booster dose (No Boost group) according to protocols approved by the institutional review boards at the three participating sites. From the main study cohort, samples for the current analyses were available for 22 participants who had received prime and booster vaccine doses at 18 months apart and for 34 adults who had received only one priming dose. Samples from each individual were collected before vaccination (month 0), after the first vaccination (1, 3, 6, 12 and 18 months), after the second vaccination (19, 24, 30 and 36 months) and 1, 3, 6, 12, 18, 19, 24, 30 and 36 months after single vaccination in the ‘No Boost’ cohort.

### Ethics statements for human study

As described previously, human use protocols for collecting samples from the phase 2 clinical study were reviewed and approved by the institutional review boards at each participating site, including at National Institute of Allergy and Infectious Disease, National Institutes of Health (ClinicalTrials.gov ID: NCT02788227)^27^. Written informed consent was obtained from all volunteers before enrollment. Samples were anonymous, and permission to test these deidentified samples in different antibody assays was obtained from the US FDA’s Research Involving Human Subjects Committee under exemption protocol 15-064B. The study at CBER, FDA, was conducted with deidentified samples under Research Involving Human Subjects exemption 15-064B, and all assays performed fell within the permissible usages in the original consent.

### Recombinant proteins

Recombinant EBOV-GPs from Kikwit and Makona strains used in this study were purchased from Sino Biologicals. The recombinant EBOV-GP for staining B cells (EBOV-GPΔmuc_FT_Avi) was constructed with the human CD5 signal peptide sequence, followed by the coding region of GP residues 32–646 derived from the Zaire strain (GenBank: AAG40168.1) with a deletion of the mucin-like domain (residues 312–462) and a C-terminal modification cassette including a foldon trimerization motif, an enterokinase cleavage site, a 6×His tag and an Avi-Tag. The construct was codon optimized for mammalian expression and cloned into the Twist CMV β-globin expression vector using EcoRI and XbaI restriction sites. EBOV-GPΔmuc_FT_Avi was expressed by transient co-transfection with the human furin plasmid^57^ at a 2:1 mass ratio into Expi293F cells (Thermo Fisher Scientific) using ExpiFectamine 293 Transfection Reagent, according to the manufacturer’s instructions. Proteins were purified by cOmplete His-Tag Purification Resin (Roche), followed by size-exclusion chromatography on a Superpose 6 10/300 GL column (Cytiva). For staining of B cells, the purified EBOV-GP containing the Avi-Tag was site specifically biotinylated using a BirA Biotin-Protein Ligase Standard Reaction kit (Avidity), according to the manufacturer’s instructions.

### EBOV PRNT

PRNT against the homologous rVSV∆G-ZEBOV-GP vaccine virus expressing the GP of the Kikwit strain was performed as described previously^30^. Briefly, heat-inactivated (56 °C for 30 min) serum was diluted from 1:5 to 1:10,240 and mixed with an equal volume of diluted rVSV∆G-ZEBOV-GP vaccine virus for final dilutions of 1:10 to 1:20,480 and incubated for 20 h at 2–8 °C. The virus antibody was added to Vero cells for 60 min at 37 °C, followed by a methylcellulose overlay and incubation at 37 °C for 2 days. Plaques were visualized by crystal violet stain, and 60% reduction in viral plaques was determined in the presence of serum compared to that of the virus control without serum to calculate 60% neutralizing titer by linear regression. If a titer was less than the lower limit of detection (<20), it was considered 10 for data representation and statistical analyses. Multiple studies were performed to select the 60% plaque reduction endpoint-based robustness of the assay and statistical evaluation of repeatability and precision^30^.

### Affinity selection of EBOV-GP GFPDL phages with rVSV∆G-ZEBOV-GP postvaccination polyclonal human sera

GFPDL construction using the envelope GP gene of EBOV Kikwit strain in a gIII display-based phage vector (fSK-9-3) was performed as described previously^34,58^. Before panning of GFPDL with polyclonal serum antibodies, serum components that could nonspecifically interact with phage proteins were removed by incubation with UV-killed M13K07 phage-coated Petri dishes. Equal volumes of pooled polyclonal human sera from each cohort were used for each round of GFPDL panning. All samples in the ‘Prime–Boost’ (*N* = 15) and ‘No Boost’ (*N* = 23) groups were pooled for GFPDL analysis. Subsequent GFPDL affinity selection was performed in-solution (with Protein A/G) as previously described^34,58^. The GFPDL affinity selection data were acquired in duplicate (two independent experiments by laboratory personnel, who were blinded to sample identity), and similar numbers of phage clones and epitope repertoires were observed in both phage display analyses.

### B cell analyses

Anti-EBOV-GP IgG titers measured by ELISA in the phase 2 trial and previously reported^27^ were used in the current study for comparison with B cells. Cryopreserved PBMCs were thawed and stained with a 28-color panel to phenotype B cells and measure their EBOV-GP specificity by spectral flow cytometry (Extended Data Table 3), as described previously^59^, with the following modifications. The EBOV-GP trimer was biotinylated and tetramerized with two fluorescently labeled streptavidin (SA; SA-BV421 and SA-Alexa Fluor 488) at a molar ratio of 4:1. The stained and fixed cells were acquired on an Aurora spectral cytometer using SpectroFlo Software v3.2.1 (Cytek Biosciences) and analyzed using FlowJo v10.10 (BD Biosciences). FCS files from gated single, live CD45^+^CD3^−^CD19^+^ B cells were exported from all participants at all time points for downstream analysis.

FlowSOM clustering and UMAP embedding were performed on exported FCS files using the OMIQ platform (omiq.ai) as previously described^59^. The following markers were used to perform FlowSOM clustering on a total of 13 million CD19^+^ cells: CD19, CD20, CD10, CD38, CD27, CD21, CD71, CD11c, FCRL5, CXCR3, CD95, CD11a, CD45RB, CD39, CD29, CD73, IL-4R, CD23, CD200, IgD, IgM, IgG and IgA, with the number of desired clusters set to 30. EBOV-GP-specific B cells were identified in each cluster by gating on EBOV-GP BV421 and Alexa Fluor 488 double-positive cells (Extended Data Fig. 2a). The raw data generated by FlowSOM clustering were exported as a .csv file from OMIQ for further analysis.

### Binding of rVSV∆G-ZEBOV-GP-vaccinated human sera to recombinant GP and off-rate measurements by SPR

Steady-state equilibrium binding of all serum samples from rVSV∆G-ZEBOV-GP-vaccinated individuals was monitored at 25 °C using a ProteOn SPR (Bio-Rad), as described before^23,31^. Briefly, purified recombinant GP was coupled to a GLC sensor chip via amine coupling with 500 RU in the test flow channels. The protein density on the chip was optimized to measure only monovalent antibody interactions. Serum diluted at 10-fold and 100-fold in PBS pH 7.4 buffer with Tween-20 and bovine serum albumin was injected at a flow rate of 50 µl min^−1^ (240-s contact duration) for association, and disassociation was performed over a 1,200-s interval. Responses from the protein surface were corrected for the response from a mock surface and for responses from a buffer-only injection. The maximum RU data shown in figures is for tenfold dilution of each serum sample. Antibody isotype analysis for the GP-bound antibodies in the postvaccination polyclonal sera was performed using SPR. Total antibody binding and antibody isotype analyses were calculated with Bio-Rad ProteOn manager software (version 3.0.1). All SPR experiments were performed twice, and the researchers performing the assay were blinded to sample identity. In these optimized SPR conditions, the variation for each sample in duplicate SPR runs was <7%.

Antibody off-rate constants, which describe the stability of the complex, that is, the fraction of complexes that decays per second, were determined directly for each postvaccination human polyclonal serum sample interaction with recombinant GP protein using SPR signal of the samples between 5 and 100 RU by the Bio-Rad ProteOn manager software for the heterogeneous sample model.

### EBOV-GP FcγR binding assay

FcγR binding assays were performed as described previously^60^. Briefly, EBOV Kikwit GP was conjugated to magnetic beads using a Bio-Plex amine coupling kit (Bio-Rad), as per the manufacturer’s instructions. Coupled beads were mixed in assay buffer in 50 µl per well in 96-well flat-bottom plates, and serum samples were added to each well at a 100-fold dilution and incubated for 1 h at room temperature. Beads coupled to human serum albumin were included as a control. After incubation, plates were washed, and biotinylated human FcγRs or C1q proteins were added, followed by incubation with Bio-Plex Streptavidin-PE. Data were acquired using a Bio-Plex 200 system. Each sample was run in duplicate, and all data were normalized by subtracting values of human serum albumin.

### ADCC and ADCP assays

ADCC and ADCP assays were performed on the samples with Promega kits G7015 and G9901, respectively. HEK293T cells were transfected with plasmid expressing GP from the EBOV Kikwit strain using Thermo Fisher’s Lipofectamine 3000. After 24 h of incubation, the cells were then resuspended and counted to seed 1.25 × 10^4^ cells per well in 96-well plates. One day later, GP expression was confirmed on the transfected cell surface before the start of ADCC/ADCP assays. For ADCC/ADCP assays, GP-transfected HEK293T target cells were incubated for 30 min with the human samples in triplicate at room temperature, which was then aspirated before the addition of 7.5 × 10^4^ effector cells per well from a Promega kit at a 1:6 ratio. The plates were incubated for 6 h at 37 °C, and then Bio-Glo Luciferase Assay Reagent was added to the wells. Luminescence was then measured and reported as relative light units.

### Statistical analyses

Analyses were performed using R software, v4.5.1 (R Foundation for Statistical Computing). Statistical differences between and within the two groups were determined using a paired or Welch’s two-sample *t*-test, respectively. Although the outcome used for analysis was usually obvious from context, we clarify the outcome in two of the analyses. For the month 19 between group comparison in Fig. 1d, the change from month 18 to month 19 was used as the outcome. For the comparison of isotypes in Fig. 4, the percentage of a given isotype (for example, IgG) was used as the outcome. Correlation coefficients were calculated using Pearson’s method and compared between the two groups using a Fisher’s *z* transformation. *P*values less than 0.05 were considered significant.

### Reporting summary

Further information on research design is available in the Nature Portfolio Reporting Summary linked to this article.

## Online content

Any methods, additional references, Nature Portfolio reporting summaries, source data, extended data, supplementary information, acknowledgements, peer review information; details of author contributions and competing interests; and statements of data and code availability are available at 10.1038/s41590-026-02459-w.

## Supplementary information

Reporting Summary

## Source data

Source Data Fig. 1 All source data.

Source Data Fig. 3 All source data.

Source Data Fig. 4 All source data.

Source Data Fig. 5 All source data.

Source Data Fig. 6 All source data.

Source Data Extended Data Fig. 1 All source data.

Source Data Extended Data Fig. 2 All source data.

Source Data Extended Data Fig. 3 All source data.

Source Data Extended Data Fig. 4 All source data.

Source Data Extended Data Fig. 5 All source data.

Source Data Extended Data Fig. 6 All source data.

Source Data Extended Data Fig. 7 All source data.

## Figures and Tables

**Extended Data Table 1:**
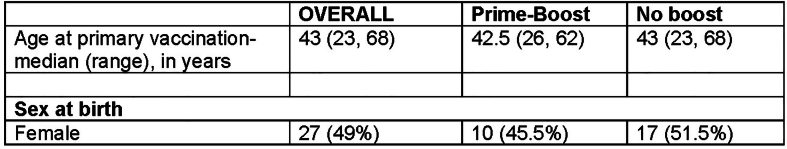
Summary of Participants demographics

**Extended Data Table 2:**
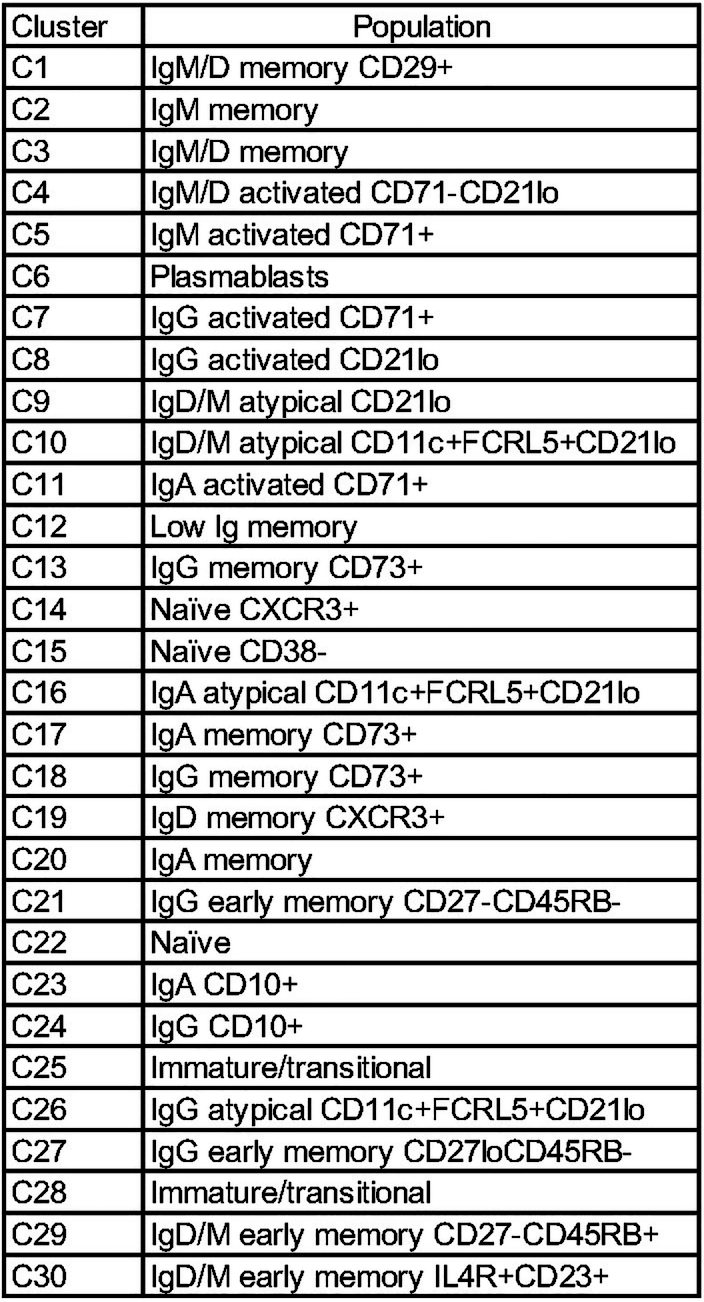
Cluster annotations

**Extended Data Table 3:**
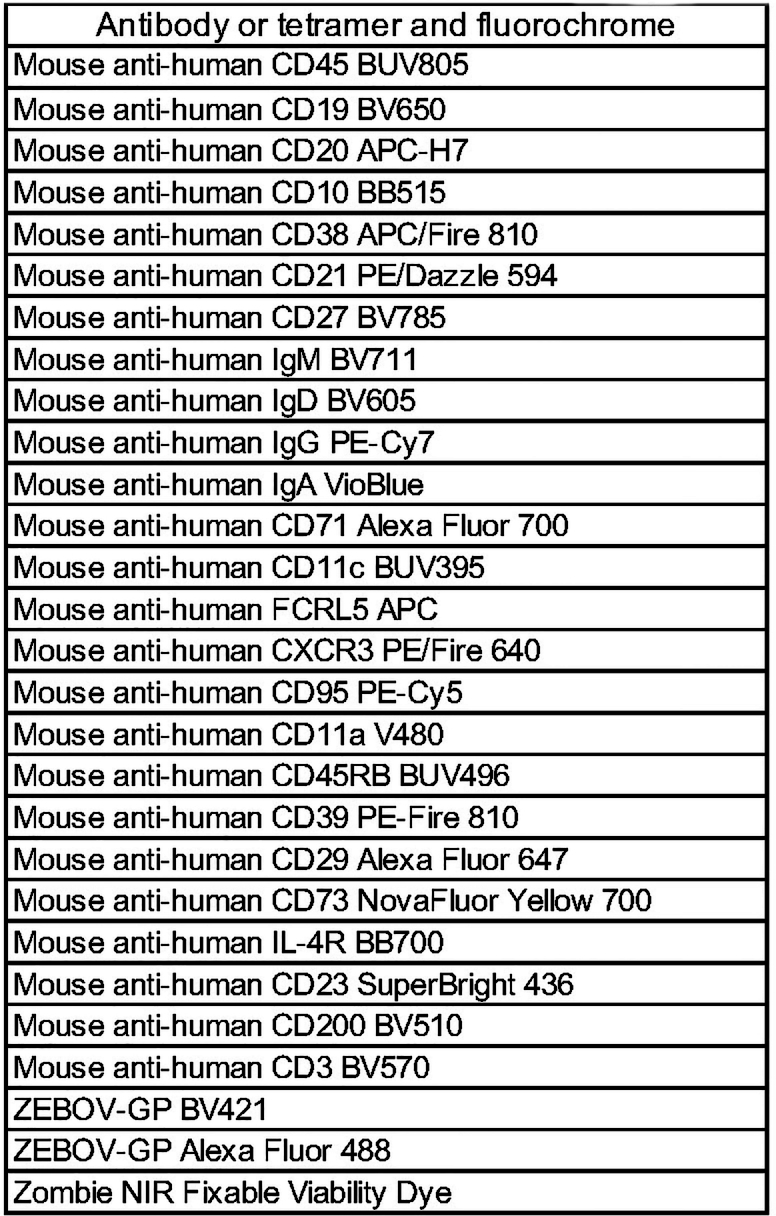
28-color flow cytometry panel

## Data Availability

All data are shown in the manuscript figures and Extended Data files. There are restrictions to the availability of the GFPDL technology and the GFPDL data described in this study (Fig. 2), due to a US patent application. The sequencing data can be made available on request from the corresponding author (S.K.) upon signing of appropriate agreements, as required for a patented invention. Source data are provided with this paper.

## Code availability

No custom code was used in this study.
