# Supplementary material for: Improved VSV-Ebola-GP booster vaccination approach promotes antibody affinity maturation and durable anti-Ebola immunity in humans
Source: Nat Immunol. 2026 Mar 19;27(5):1053–65. doi: 10.1038/s41590-026-02459-w (PMC13132720; doi:10.1038/s41590-026-02459-w)
Supplement: Supplementary file 1 — Reporting Summary [file 41590_2026_2459_MOESM1_ESM.pdf]

nature portfolio

Corresponding author(s): Surender Khurana

Last updated by author(s): Feb 6, 2026

Reporting Summary

Nature Portfolio wishes to improve the reproducibility of the work that we publish. This form provides structure for consistency and transparency in reporting. For further information on Nature Portfolio policies, see our Editorial Policies and the Editorial Policy Checklist.

Statistics

For all statistical analyses, confirm that the following items are present in the figure legend, table legend, main text, or Methods section.

n/a	Confirmed	
☐	☒	The exact sample size (*n*) for each experimental group/condition, given as a discrete number and unit of measurement
☐	☒	A statement on whether measurements were taken from distinct samples or whether the same sample was measured repeatedly
☐	☒	The statistical test(s) used AND whether they are one- or two-sided<br>*Only common tests should be described solely by name; describe more complex techniques in the Methods section.*
☐	☒	A description of all covariates tested
☐	☒	A description of any assumptions or corrections, such as tests of normality and adjustment for multiple comparisons
☐	☒	A full description of the statistical parameters including central tendency (e.g. means) or other basic estimates (e.g. regression coefficient) AND variation (e.g. standard deviation) or associated estimates of uncertainty (e.g. confidence intervals)
☐	☒	For null hypothesis testing, the test statistic (e.g. *F*, *t*, *r*) with confidence intervals, effect sizes, degrees of freedom and *P* value noted<br>*Give P values as exact values whenever suitable.*
☒	☐	For Bayesian analysis, information on the choice of priors and Markov chain Monte Carlo settings
☒	☐	For hierarchical and complex designs, identification of the appropriate level for tests and full reporting of outcomes
☐	☒	Estimates of effect sizes (e.g. Cohen's *d*, Pearson's *r*), indicating how they were calculated

Our web collection on statistics for biologists contains articles on many of the points above.

Software and code

Policy information about availability of computer code

Data collection	Antibody data was collected in MS Excel version 16.57. BioRad ProteON Manager software (Version 3.1.0) was used to collect antibody binding data from SPR machine (www.Biorad.com). B-cell data was collected using SpectroFlo software (version 3.2.1).
Data analysis	Antibody titers were calculated using Prism 9.3.1 (GraphPad Software). BioRad ProteON Manager software (Version 3.1.0) for antibody binding analysis from SPR machine (www.Biorad.com). B-cell data analysis was performed using FlowJo (version 10.10), OMIQ platform (www.omiq.ai) with UMAP and FlowSOM algorithms. Statistical differences between and within the two groups were determined using paired or Welch's two-sample t-test, respectively. Correlation coefficients were calculated using the Pearson's method and compared between the two groups using a Fisher's z-transformation. All information is provided the statistical methods section.

For manuscripts utilizing custom algorithms or software that are central to the research but not yet described in published literature, software must be made available to editors and reviewers. We strongly encourage code deposition in a community repository (e.g. GitHub). See the Nature Portfolio guidelines for submitting code & software for further information.

Data

Policy information about availability of data

All manuscripts must include a data availability statement. This statement should provide the following information, where applicable:

- Accession codes, unique identifiers, or web links for publicly available datasets
- A description of any restrictions on data availability
- For clinical datasets or third party data, please ensure that the statement adheres to our policy

All data are shown in the manuscript figures and Extended Data file. Source data are provided with alongside this paper as 'Source Data' file. There are restrictions to the availability of the GFPDL technology and the GFPDL data described in this study (Figure 2), due to US patent application. The sequencing data can be made available on request from corresponding author (S.K.) upon signing of appropriate agreements, as required for patented invention.

Research involving human participants, their data, or biological material

Policy information about studies with human participants or human data. See also policy information about sex, gender (identity/presentation), and sexual orientation and race, ethnicity and racism.

Reporting on sex and gender	All adults irrespective of sex were eligible for the study. Sex and/or gender was considered in the study design and was determined based on self-report by the study participants. All participants reported to be binary (either Male or Female). Study findings apply to adults of both sexes. No sex-based analyses were performed in this study. The clinical trial was performed in healthy adults with equal distribution of males and females. The consent was not obtained for reporting and sharing individual-level data. No sex/gender-based analyses have been performed.
Reporting on race, ethnicity, or other socially relevant groupings	All adults irrespective of their race or ethnicity were eligible for the study. Race or ethnicity was not considered in the study design and study findings apply to adults of all race or ethnicities. No race-based analyses were performed in this study.
Population characteristics	Participants in this study were aged 18-65 years old. All adults irrespective of sex, race, ethnicity, or previous health status who provided informed consent were eligible for the different clinical study as described in materials and methods. Sex, race, ethnicity, or previous health status was not considered in the study design and study findings apply to all adults of both sexes or any race or ethnicity. No sex-based or race-based analyses were performed in this study.
Recruitment	All healthy adult participants were eligible without any specific selection criteria and no selection bias or any other apparent bias. Samples were collected from adults following informed consent to participate in the clinical trials. Adult participants were recruited and enrolled in the Ebola vaccine trial as per IRB approval.
Ethics oversight	Healthy adults (18-65 years) were enrolled into an IRB-approved clinical study protocol following informed consent as described in study design.<br>Vaccinated samples were obtained from participants in randomized, placebo controlled, prime-boost, phase II clinical trial performed in healthy adults >18 years old at risk for potential occupational exposure to EBOV that was reviewed and approved by the institutional review boards at each participating site, including at NIAID, National Institutes of Health (ClinicalTrials.gov ID: NCT02788227)<br>Samples were anonymous, and permission to test these de-identified samples in different antibody assays was obtained from the U.S. Food and Drug Administration's Research Involving Human Subjects Committee (FDA-RIHSC) under exemption protocol #15-064B.

Note that full information on the approval of the study protocol must also be provided in the manuscript.

Field-specific reporting

Please select the one below that is the best fit for your research. If you are not sure, read the appropriate sections before making your selection.

☒ Life sciences ☐ Behavioural & social sciences ☐ Ecological, evolutionary & environmental sciences

For a reference copy of the document with all sections, see nature.com/documents/nr-reporting-summary-flat.pdf

Life sciences study design

All studies must disclose on these points even when the disclosure is negative.

Sample size	All available samples were analyzed in this study
Data exclusions	No data was excluded
Replication	Neutralization, GFPDL,FcR and SPR experiments were performed twice on each sample by independent researchers in the lab. The replications were successful. The variation in duplicate experimental runs was less than 2-fold (one-serial dilution) for neutralization. The variation in duplicate SPR experimental runs was <7% and 8% for FcR assay. Given limited availability of cells, B-cell experiments were

	performed once, although reproducibility was confirmed with duplicate samples in both groups at different time-points based on sample availability.
Randomization	All samples from the adults were analyzed in this study. The vaccine clinical trial was blinded and randomized performed in adults. Initially, no patient information was provided, and all the immune analyses were conducted blindly by the researcher's performing the assays.
Blinding	Experiments were performed by different investigators, who were blinded to sample identity.

Reporting for specific materials, systems and methods

We require information from authors about some types of materials, experimental systems and methods used in many studies. Here, indicate whether each material, system or method listed is relevant to your study. If you are not sure if a list item applies to your research, read the appropriate section before selecting a response.

Materials & experimental systems

n/a	Involved in the study
☐	☒ Antibodies
☐	☒ Eukaryotic cell lines
☒	☐ Palaeontology and archaeology
☒	☐ Animals and other organisms
☒	☐ Clinical data
☒	☐ Dual use research of concern
☒	☐ Plants

Methods

n/a	Involved in the study
☒	☐ ChIP-seq
☐	☒ Flow cytometry
☒	☐ MRI-based neuroimaging

Antibodies

Antibodies used

Donkey anti-human IgG-Fc specific antibody (Cat # 709-005-098), Donkey anti-human IgM Fc5u specific (Cat # 709-005-073), goat anti-human serum IgA alpha chain specific (Cat # 109-005-011) were purchased from Jackson Immuno Reseearch.
1. CD45 BUV805, HI30, BD Biosciences, catalog #612891
2. CD19 BV650, SJ25-C1, BD Biosciences, catalog #563226
3. CD10 BB515, HI10a, BD Biosciences, catalog #564638
4. CD11c BUV395, B-ly6, BD Biosciences, catalog #563787
5. CD20 APC-H7, 2H7, BD Biosciences, catalog #560734
6. IgG PE-Cy7, G18–145, BD Biosciences, catalog #561298
7. CD95 PE-Cy5, DX2, Biolegend, catalog #305610
8. CD3 BV570, UCHT1, Biolegend, catalog #300436
9. IgD BV605, IA6–2, Biolegend, catalog #348232
10. IgM BV711, MHM-88, Biolegend, catalog #314540
11. CD27 BV785, O323, Biolegend, catalog #302832
12. CD21 PE/Dazzle 594, BU32, Biolegend, catalog #354922
13. CD38 APC/Fire 810, HB-7, Biolegend, catalog #356644
14. CD71 Alexa Fluor 700, GY1G4, Biolegend, catalog #334130
15. CXCR3 PE-Fire 640, G025H7, Biolegend, catalog #353764
16. CD39 PE-Fire 810, A1, Biolegend, catalog #328245
17. CD45RB BUV496, MT4, Biolegend, catalog #750194
18. CD29 Alexa Fluor 647, TS2/16, Biolegend, catalog #303018
19. CD73 NovaFluor Yellow 700, AD2, ThermoFisher, catalog #H016T03Y06
20. IgA VioBlue, IS11-8E10, Miltenyi Biotec, catalog #130-113-479
21. FCRL5 APC, 509f6, Biolegend, catalog #340306
22. CD11a BV480, HI111, BD Biosciences, catalog #746551
23. IL-4R BB700, hIL4R-M57, BD Biosciences, catalog #745925
24. CD200 BV510, MRC OX-104, BD Biosciences, catalog #563254
25. CD23 SuperBright 436, EBVCS2, ThermoFisher, catalog #62-0238-42

Validation

Isotyping antibodies from Jackson Immuno Reseearch were produced, tested and validated by the manufacturer.
1. CD45 BUV805; HI30, Reactivity: Human (QC testing, BD Biosciences); Application: Flow cytometry (Routinely Tested, BD Biosciences)
2. CD19 BV650; Reactivity: Human (QC testing, BD Biosciences); Application: Flow cytometry (Routinely Tested, BD Biosciences)
3. CD10 BB515; Reactivity: Human (QC testing, BD Biosciences); Rhesus, Cynomolgus, Baboon (Tested in Development, BD Biosciences); Application: Flow cytometry (Routinely Tested, BD Biosciences)
4. CD11c BUV395; Reactivity: Human (QC testing, BD Biosciences); Application: Flow cytometry (Routinely Tested, BD Biosciences)
5. CD20 APC-H7; Reactivity: Human (QC testing, BD Biosciences); Rhesus, Cynomolgus, Baboon (Tested in Development, BD Biosciences); Application: Flow cytometry (Routinely Tested, BD Biosciences)
6. IgG PE-Cy7, Reactivity: Human (QC testing, BD Biosciences); Application: Flow cytometry (Routinely Tested, BD Biosciences)
7. CD95 PE-Cy5, Reactivity: Human, Cynomolgus, Rhesus (BioLegend); Application: Flow cytometry (Quality tested, BioLegend)
8. CD3 BV570; Reactivity: Human (BioLegend); Cross-reactivity: Chimpanzee (BioLegend); Application: Flow cytometry (Quality tested, BioLegend)
9. IgD BV605; Reactivity: Human (BioLegend); Application: Flow cytometry (Quality tested, BioLegend)
10. IgM BV711; Reactivity: Human, African Green, Baboon, Cynomolgus, Rhesus (BioLegend); Application: Flow cytometry (Quality

tested, BioLegend)
11. CD27 BV785; Reactivity: Human, African Green, Baboon, Cynomolgus, Rhesus, Squirrel Monkey (BioLegend); Application: Flow cytometry (Quality tested, BioLegend)
12. CD21 PE/Dazzle 594, Reactivity: Human, African Green, Baboon, Cynomolgus (BioLegend); Application: Flow cytometry (Quality tested, BioLegend)
13. CD38 APC/Fire 810, Reactivity: Human (BioLegend); Application: Flow cytometry (Quality tested, BioLegend)
14. CD71 Alexa Fluor 700, Reactivity: Human (BioLegend); Application: Flow cytometry (Quality tested, BioLegend)
15. CXCR3 PE-Fire 640, Reactivity: Human, Cynomolgus, Rhesus, African Green, Baboon (BioLegend); Application: Flow cytometry (Quality tested, BioLegend)
16. CD39 PE-Fire 810, Reactivity: Human, Cynomolgus, Rhesus (BioLegend); Application: Flow cytometry (Quality tested, BioLegend)
17. CD45RB BUV496, Reactivity: Human (Tested in Development, BD Biosciences); Application: Flow cytometry (Qualified, BD Biosciences)
18. CD29 Alexa Fluor 647, Reactivity: Human, African Green, Baboon, Cynomolgus, Dog, Horse, Rhesus (BioLegend); Application: Flow cytometry (Quality tested, BioLegend)
19. CD73 NovaFluor Yellow 700, Human (ThermoFisher); Application: Flow cytometry (ThermoFisher)
20. IgA VioBlue; Reactivity: Human (QC tested, Miltenyi Biotec); Application: Flow cytometry (Miltenyi Biotec)
21. FCRL5 APC, Reactivity: Human (BioLegend); Application: Flow cytometry (Quality tested, BioLegend)
22. CD11a BV480, Reactivity: Human (Tested in Development, BD Biosciences); Application: Flow cytometry (Qualified, BD Biosciences)
23. IL-4R, Reactivity: Human (Tested in Development, BD Biosciences); Application: Flow cytometry (Qualified, BD Biosciences)
24. CD200 BV510, Reactivity: Human (QC testing, BD Biosciences); Application: Flow cytometry (Routinely Tested, BD Biosciences)
25. CD23 SuperBright 436, Human (ThermoFisher); Application: Flow cytometry (ThermoFisher)

Eukaryotic cell lines

Policy information about cell lines and Sex and Gender in Research

Cell line source(s)	Vero E6 cell line was obtained from ATCC (Catalog number #ATCC-CRL-1586). Cell lines used in ADCC and ADCP assays were obtained from Promega (G7015 and G9901)
Authentication	The cell line was authenticated by karyotyping or other genomic techniques by ATCC or by Promega.
Mycoplasma contamination	Negative for Mycoplasma
Commonly misidentified lines (See ICLAC register)	No misidentified cell lines were used in the study.

Flow Cytometry

Plots

Confirm that:

☒ The axis labels state the marker and fluorochrome used (e.g. CD4-FITC).

☒ The axis scales are clearly visible. Include numbers along axes only for bottom left plot of group (a 'group' is an analysis of identical markers).

☒ All plots are contour plots with outliers or pseudocolor plots.

☒ A numerical value for number of cells or percentage (with statistics) is provided.

Methodology

Sample preparation	Cryopreserved peripheral blood mononuclear cells (PBMCs) were thawed and stained for flow cyteometry
Instrument	Cytek Aurora (5 lasers)
Software	Data were acquired using the SpectroFlo Software version 3.2.1 (Cytek Biosciences) and analyzed using FlowJo version 10.10.0, and OMIQ platform (www.omiq.ai)
Cell population abundance	We collected on average 76,000 B lymphocytes for each sample.
Gating strategy	The cells were gated as follows: Single cells identified by FSC-H/FSC-A, followed by CD45+ lymphocytes (CD45/SSC-A), followed by the dead cell exclusion using Zombie NIR stain. B lymphocytes were identified as CD19+CD3-, exported and used for high-dimensional analysis. EBOV-GP specific B cells were identified by gating on EBOV-GP BV421 and AF488 double positive cells

☒ Tick this box to confirm that a figure exemplifying the gating strategy is provided in the Supplementary Information.
